# Validation of the short version of the obsessive compulsive spectrum questionnaire

**DOI:** 10.3389/fpsyg.2023.1157636

**Published:** 2023-06-27

**Authors:** Liliana Dell’Osso, Benedetta Nardi, Chiara Bonelli, Davide Gravina, Francesca Benedetti, Luca Del Prete, Gabriele Massimetti, Giulia Amatori, Barbara Carpita, Ivan Mirko Cremone

**Affiliations:** Department of Clinical and Experimental Medicine, University of Pisa, Pisa, Italy

**Keywords:** obsessive–compulsive spectrum, OCD, spectrum model, questionnaire, Obsessing-compulsive disorder

## Abstract

**Aim:**

In the recent years, a rising amount of research has stressed the importance of a dimensional perspective on mental disorders. In particular, the conceptualization of an obsessive–compulsive spectrum appears to be in line with the very first descriptions of Obsessive–Compulsive Disorder and has been partially acknowledged by the inclusion of the “OCD-spectrum related syndromes and disorders” section in the DSM-5. The goal of the current study is to ascertain the psychometric characteristics of the Obsessive–Compulsive Spectrum–Short Version (OBS-SV), a novel questionnaire designed to measure the complete range of obsessive–compulsive symptoms, from severe full blown to subthreshold ones.

**Methods:**

Forty three subjects with a clinical diagnosis of OCD according to the Diagnostic and Statistical Manual of Mental Disorders (DSM-5); 42 subjects with a clinical diagnosis of social anxiety disorder (SAD), and 60 individuals without current or lifetime mental disorders (HC) were recruited from the Psychiatric Clinic of the University of Pisa. Subjects were assessed with the SCID-5, the Yale Brown Obsessive Compulsive Scale (Y-BOCS) and the OBS-SV.

**Results:**

OBS-SV showed strong test–retest reliability for both the total and the domains scores, as well as a high level of internal consistency. The Pearson’s coefficients for the OBS-SV domain scores ranged from 0.771 to 0.943, and they were positively and strongly linked with one another (*p* < 0.001). The OBS-SV total score had a strong correlation with each of the OBS-SV domain scores. All correlation coefficients between OBS-SV and additional measures of OCS were observed to be strong, significant and positive. Both OBS-SV domain and overall score differences between diagnostic groups were found to be statistically significant. From HCs, to the SAD, up to the OC group, which had the highest values, the OBS-SV total score grew dramatically and progressively.

**Conclusion:**

The OBS-SV demonstrated significant convergent validity with other dimensional OCD measures, excellent internal consistency, and test–retest reliability. Across the three diagnostic categories, the questionnaire functioned differently, with a rising score gradient from healthy controls through SAD patients to OCD subjects.

## 1. Introduction

The Obsessive–Compulsive Disorder (OCD) is a severe chronic illness that affects a significant portion of the world’s population and, also due its limited response to pharmacological treatments, often represent one of the major management challenges for clinical psychiatrists (Karno et al., 1988). OCD is characterized by the presence of obsessions and compulsions; although the presence of only one of these two symptoms is sufficient for receiving an OCD diagnosis, patients typically display both of them (American Psychiatric Association, 2013, 2023). Obsessions are persistent and recurrent thoughts, impulses, or urges that makes the individual feel uncomfortable or anxious. These can be differentiated from delusions since they are frequently perceived as intrusive or ego-dystonic, and they are typically recognized as unrealistic or exaggerated, although the difference can become hazy in some circumstances. Compulsions, on the other hand, are repetitive behaviors or mental acts carried out with the aim to prevent dreaded events or reduce anxiety and distress, frequently in response to obsessions (American Psychiatric Association, 2013, 2023). A recent national survey highlighted that OCD has a lifetime prevalence of 2–3% and it is often associated with other comorbid mental disorders, although prevalence rates may vary depending on specific regional areas (Fontenelle et al., 2006; Kessler et al., 2012). Despite the majority clinical researches on OCD have focused on the full-blown condition (Grant et al., 2012), the literature is stressing how, like many other psychiatric disorders, OCD can also occur in a milder subsyndromal form: in light of a dimensional approach to psychopathology, subthreshold obsessive–compulsive (OC) traits can be found continuously distributed in the general population (Chamberlain et al., 2005; Grant and Chamberlain, 2019). In addition, OC traits have been observed in clinical population of patients with different psychiatric conditions, such as autism spectrum disorder (Postorino et al., 2017; Pazuniak and Pekrul, 2020), mood disorders (Tonna et al., 2021), feeding and eating disorders (Amianto et al., 2022; Reilly et al., 2022), personality disorders (McKay et al., 2000) and even catatonic manifestations (Mukai et al., 2011).

The main international classifications of mental disorders, the Diagnostic and Statistical Manual of Mental Disorders (DSM) and the International Classification of Diseases (ICD), in their most recent version successfully focused on increasing the specificity of psychiatric nomenclature. However, due the rigidity of the categorical system failed to adequately represent the full spectrum of potentially distressing psychopathological symptoms with which the patient may come to clinical attention. This spectrum of manifestation comprehends a wide range of subclinical, atypical, prodromal or residual symptoms and traits that may have an impact on subject’s quality of life although not being sufficient for receiving a full-blown diagnosis (Angst et al., 1997; Pincus et al., 1999; Helmchen and Linden, 2000).

A spectrum model of psychopathology is more useful for identifying the broad sub-threshold conditions that may coexist with the main psychiatric diseases (Frank et al., 1998). Traditionally, in this framework the word “spectrum” has been employed to emphasize the connection between different symptomatic clusters or between different levels of severity of a same disorder (Akiskal and Akiskal, 1992) or different disorders (Hollander and Wong, 1995). Starting from these considerations, at the beginning of the current century was developed the “Collaborative Spectrum Project” with the aim to describe and better characterize the subtle and atypical signs linked to numerous DSM disorders (Cassano et al., 1997, 1998, 1999; Dell’Osso et al., 2003, 2016a,b, 2017, 2022, 2023; Carmassi et al., 2023). According to the spectrum project conceptualization (Cassano et al., 1997, 1998, 1999; Dell’Osso et al., 2003, 2016b, 2017, 2022, 2023, Carmassi et al., 2023), the spectrum of a disorder encompasses both full-blown and prototypical presentations along with sub-clinical and non-typical ones. It also comprises discrete signs and symptoms, symptom clusters, behavioral patterns, temperamental and/or personality qualities, and symptoms that are assumed to be related to the primary symptoms of the disorder. These last manifestations—signs, symptoms, and behavioral patterns—might be regarded as prodromes of a condition that has not yet fully manifested, or as aftereffects of a previously experienced, full-blown disorder, furthermore, they may also persist throughout life without fully manifesting clinically (Dell’Osso et al., 2000). In particular, the conceptualization of an OC spectrum appears to be in line even with some of the first conceptualizations of the disorder, when Janet described, under the term of psychasthenia, a picture that ranged from subthreshold obsessive features to the “obsessional personality” and “obsessional neurosis” (Gardiner, 1904) concept later resumed by Hollander et al. with the elaboration of the “OCD-spectrum related syndromes and disorders” (Hollander and Wong, 1995). Moreover, currently, the clinical evidence of OC-related disorders and the recognition of over-threshold and subthreshold OC comorbidity are compatible with the spectrum model of OC spectrum symptoms along an obsessive–compulsive component (Dell’Osso et al., 2000).

In the context of the spectrum project, together with other spectrum based psychometric instruments, the “Obsessive–Compulsive Spectrum Self-report” (OBS-SR) questionnaire was developed and validated with the aim to assess not only the prototypic symptoms of OCD but also unusual manifestations, temperamental traits, and other noteworthy clinical and sub-clinical aspects linked to the main symptoms (Dell’Osso et al., 2000, 2002). The questionnaire demonstrated a moderate internal consistency and a good inter-rater reliability along with a good discriminant validity; however, its use in the daily clinical practice remained quite difficult due to the long time needed to fill out it, which is around 50 min. In addition, the instrument, being developed in the early 2000s, still includes dated and redundant items.

As the main authors of the OBS-SR, we aimed to develop a new revised and shortened version of the questionnaire, the Obsessive–Compulsive Spectrum – Short Version (OBS-SV), which should be characterized by a reduced compilation time, but also by a greater internal consistency, inter-rater reliability and discriminant validity, also excluding dated items and including more modern ones, useful for the clinical practice and the investigation of both the full-blown and the milder subsyndromal form and OCD traits. In particular, the OBS-SV evaluates not only the typical OCD symptoms but also uncommon presentations, temperamental characteristics, and other notable clinical features related to the primary symptoms. Moreover, compared to the other commonly available measures of OCD, the OBS-SV is able to detect and evaluate the presence of obsessive traits in patients suffering from other mental disorder and in the non-clinical population, allowing the clinician to better assess the clinical picture and to recognize possible risk factor for the development of a concomitant disorder. In this study we sought to validate the OBS-SV questionnaire in a clinical population of subjects with OCS, Social Anxiety Disorder (SAD) and in healthy controls (HC). In particular, considering the reported presence of sub-threshold OCD traits in subjects with SAD and the overlapping features between OCD and SAD spectra, the SAD group was recruited as a potential intermediate group for OCD traits between OCD patients and HC (Carpita et al., 2020). We chose to recruit this kind of sample, with SAD subjects as an intermediate group, guided by evidences from numerous research that showed a substantial correlation between SAD and OCD. In fact, not only the comorbidity between OCD and SAD is reported to range from 12 to 42% in clinical samples and from 15 to 43.5% in the general population (Baldwin et al., 2008; Assunção et al., 2012), but also SAD is the most frequent co-occurring anxiety disorder among OCD patients (43.5%) as well as OCD has been noted to frequently co-occur with anxiety disorders (Bartz and Hollander, 2006; Ruscio et al., 2010). Despite that, OCD has been separated from anxiety disorders both in the DSM-5 and in the ICD-11 and is now included in a different category that also includes related conditions like body dysmorphic disorder (BDD), trichotillomania, hoarding disorder, and skin-picking disorder. This approach, while emphasizing the distinctive characteristics of the obsessive–compulsive spectrum, ignores the characteristics that OCD and anxiety disorders have in common, which are instead shown by both the responsiveness to similar therapies and the commonly observed comorbidity between these diseases (Carrasco et al., 1992; Baldwin et al., 2008; Assunção et al., 2012). According to the dimensional approach, identifying potential transnosographic symptoms among psychiatric disorders may help us better understand psychopathology and eventually get past the drawbacks of the existing nosological classification, like the high rates of comorbidity. In particular, the choice of SAD as an intermediate group for obsessive traits between OCD and HC was in line with a previous study from Dell’Osso et al., which highlighted intermediate levels of OCD traits among subjects with SAD (Carpita et al., 2020).

In this framework, the aim of the study was to validate the OBS-SV questionnaire in a clinical population of subjects with OCS, SAD, HC, and to evaluate its psychometric properties, expecting the questionnaire not only to report a significant convergent validity with other dimensional OCD measures, great internal consistency, and test–retest reliability, but also to perform differently in the three diagnostic categories.

## 2. Methods

Data have been collected between September 2022 and December 2023 at the Psychiatric clinic of the University of Pisa.

### 2.1. Study sample and procedure

The sample was composed of 145 subjects which comprised into three diagnostic categories and assessed using DSM-5 diagnostic criteria. Ages under 18, language or intellectual disabilities that made it difficult to complete the examinations, mental disabilities, a lack of collaboration skills, and persistent psychotic symptoms were considered exclusion grounds. All groups were individuated as follows: 43 subjects with a clinical diagnosis of OCD; 42 subjects with a clinical diagnosis of SAD and 60 members of the medical and paramedical workforce who do not currently have or have ever had mental disorders (HC). All subjects in order to be recruited must be aged between 18 and 70 and willing to sign an informed consent. Diagnosis of OCD and SAD, and the lack of mental disorders among HC, were confirmed using the Structured Clinical Interview for DSM-5, Research Version (SCID-5-RV) (First et al., 2015). The test–retest reliability of the OBS-SV was assessed using 30 subjects randomly selected, 10 belonging to the OCD group, 10 from the SAD group and 10 HC.

A second evaluation conducted over a period of 21 days following the original assessment in order to give evidence for the temporal stability of the scores. The study was conducted in accordance with the Declaration of Helsinki. The study was fully explained to the eligible individuals, who then gave their written informed permission after having a chance to ask any questions. The subjects received no compensation for taking part.

### 2.2. Measures

Assessment procedures included the SCID-5-RV (First et al., 2015), the Yale Brown Obsessive Compulsive Scale (Y-BOCS) (Goodman et al., 1989), and the Obsessive–Compulsive Spectrum – Short Version questionnaire (OBS-SV).

The diagnostic assessment was carried out by four trained psychiatry of the university department of Psychiatry of Pisa. Raters received instruction on the obsessive compulsive spectrum and were given an extensive description of the OBS-SV. A separate group of raters was recruited to test the test–retest reliability. Raters who administered the SCID were formally certified.

#### 2.2.1. The Yale brown obsessive compulsive scale

The Y-BOCS is a clinician-rated scale that is considered by many investigators to be the gold-standard for measuring OCD symptoms severity (Pato et al., 1994; Melli et al., 2015). It consists in two sections: the first 67 items are a symptoms inventory, organized by category: the first 29 items investigate the obsessions, the second 29 the compulsions and the last 9 the presence of avoidant behavior. The next section, which consists of 10 items, is the severity scale. Its goal is to gather all the data on time spent, distress, resistance, interference, and level of control for both obsessions and compulsions. Finally, the last 4 items consider the indices of insight, reliability, global severity and global improvement. The Y-BOCS showed an overall strong internal consistency (α = 0.89), high inter-rater (ICC = 0.96) and 1-week test–retest reliability (ICC = 0.85) (Storch et al., 2010).

#### 2.2.2. The obsessive–compulsive spectrum–short version questionnaire

The OBS-SV consist of 139 items, organized in 6 domains (104 items) and 2 appendices (35 items). The answers to the different items are dichotomously coded (yes/no) and the scores for the individual domains and appendices are calculated by counting the number of positive responses. The *Doubt* domain (10 items) explore the areas of insecurity, uncertainty and indecisions related to personal feelings, emotions and behaviors. The *Hypercontrol* domain (37 items) investigates the propensity to exert control by exploring area of caution, personal accountability, checking, emotional control, other-directed control, conformity, conventional values and magical thinking. The *Temporal dimension* domain (7 items) study different ways to use time, such as compulsive slowness and hyper- or anti-economic time management. The *Perfectionism* domain (16 items) investigates the propensity of accuracy, exactness, orderliness or symmetry. The *Repetition and automation* domain (8 items) concerns the sensation of feeling forced to repeat actions, or performing repetitive gestures, albeit inconvenient and inappropriate to the context. The *Obsessive themes* domain (26 items) examines seven themes of attitudes and behaviors toward contamination, cleansing, sexuality, spirituality, existentialism and aggressiveness.

The first appendix, *Childhood and adolescence*, refers to various obsessive–compulsive characteristics emerged during infancy or adolescence, particularly at school, in ones’ free time and in the family environment. The second appendix, *Impulsivity and loss of control*, explores atypical manifestations which concern the loss of control in various context, including eating, drinking alcohol, taking drugs, and exercising excessively. As the previous version of the instrument, the OBS-SV was simultaneously developed in English and Italian language.

The scale items were created by the same authors of the already validated OBS-SR, based on the items of the previous scale. Five trained clinicians (LDO, BC, BN, DG, BC) screened the items for inclusion and disagreements were resolved by discussion. The selection of the items was based on the affinity with the clinical description given by the DMS and the recent literature; the exclusion regarded items deemed dated, not applicable to the general population due to cultural or historical factors, ambiguous or easily misunderstood or non-discriminatory for the OC spectrum. More information about the development of the previous scale, from which the final items were taken are reported elsewhere (Dell’Osso et al., 2000, 2002). Of the 183 items present in the previous version, after a process of clinical selection, only 139 items were selected. Compared to the previous version, the Doubt domain went from 13 to 10 items; the Hypercontrol domain went from 55 to 37 items; the Temporal dimension domain passed from 9 to 7 items; the Perfectionism domain went from18 to 16 items; the Repetition and automation domain went from 12 to 8 items and the Obsessive themes domain went from 33 to 26 items. The Childhood and adolescence and the Impulsivity and loss of control domains were transformed into appendix and, respectively, reduced from 23 to 18 items and from 20 to 17 items.

### 2.3. Statistical analyses

The Cronbach’s alpha was determined for each domain and the questionnaire’s overall score in order to estimate the internal consistency of the OBS-SV. To ascertain how each item affected the instrument’s accuracy, the variations in alpha with removed items were evaluated. Computing bivariate Pearson’s correlation coefficients between the six domain scores and between each domain score and the overall score allowed researchers to examine the validity of the instrument’s internal structure. By calculating the intra-class correlation coefficient (ICC) on a subgroup of 30 participants randomly selected from the original database and re-evaluated after a gap of 3 weeks, the test–retest reliability of the questionnaire was examined. By measuring the Pearson’s correlation coefficients between the OBS-SV domains and total scores as well as the Y-BOCS total score as an alternate measure of OCD, the convergent validity was examined. The mean total and domain scores recorded in the three diagnostic groups were compared by a One-way analysis of variance to examine the instrument’s discriminatory ability (Known-groups validity) (ANOVA). *Post-hoc* comparisons were made using the Bonferroni Test. With SPSS version 26.0, all statistical analyses were carried out (IBM Corp, 2019).

## 3. Results

The OC sample reported a mean age of 40.95 years (± 11.48) and consisted of 19 (44.2%) males and 24 (55.8%) females. The SAD sample reported a mean age of 40.95 years (± 13.22) and consisted of 21 (50%) males and 21 (50%) females. The HC group reported a mean age of 37.25 years (± 13.48) and consisted in 27 (45%) males and 33 (55%) females (see Table 1). Regarding the educational level, 13 (9%) subjects reported to have a master degree, 42 (29%) subjects reported to have a degree, 41 (28.3%) subjects referred to have graduated, 12 (8.3%) subjects had a middle school certificate, 1 (0.7) subject did not finish the elementary school and 1 subject preferred not to disclose. Regarding the occupational role, 19 (13.1%) subjects were students, 21 (4.5%) subjects were unoccupied, 16 (11%) subjects were housewives, 39 (26.9%) subjects were employed, 4 (2.8%) subjects were retired and 13 (9%) subjects preferred not to disclose. Regarding the marital status 21 (14.5) subjects referred to live with their parents, 31 (21.4%) subjects were married, 36 (24.8%) subjects were unmarried, 10 (6.9%) subjects were divorced, 1 (0.7%) subject was a widower and 13 (9%) subjects preferred not to disclose.

**Table 1:** Age and sex in the overall sample and comparison between diagnostic groups.

	Total *N* (%)	OCD group *N* (%)	SAD group *N* (%)	HC group *N* (%)
*Males*	67(46.2)	19 (44,2)^a^	21 (50)^a^	27 (45)^a^
*Females*	78 (53.8)	24 (55.8)^a^	21 (50)^a^	33 (55)^a^
*Age (years)*	39.42 ± 12.89	40.95 ± 11.48	40.95 ± 13.22	37.25 ± 13.48

Each subscript letter denotes a subset of categories whose column proportions do not differ significantly from each other at the 0.5 level.

The OBS-SV was reported to have a compilation time of *circa* 30 min, proving to be a more time efficient and manageable tool compared to the previous version that reported a compilation time around 50 min.

### 3.1. Internal consistency and test–retest reliability

The Cronbach alphas and ICCs for the individual domains and the total score calculated for the entire sample are displayed in Table 2. A high level of internal consistency was shown by the OBS-SV scale. All of the OBS domains’ Cronbach alpha values are good (above the value of 0.7), and the value for the scale’s overall score is excellent (α = 0.964). As evidenced by the alpha value dropping when each item was removed individually, each item had a substantial correlation with the total and contributed in some way to the scale. With all ICCs above the value of 0.90, the test–retest reliability for total and domain scores was outstanding.

**Table 2:** OBS-SV internal consistency and test–retest reliability.

OBS-SV domains	Number of items	Cronbach’s alpha	ICC
Doubt	10	0.848	0.989
Hypercontrol	37	0.908	0.996
Temporal dimension	7	0.755	0.987
Perfectionism	16	0.847	0.993
Repetition and automation	8	0.796	0.981
Obsessive themes	26	0.895	0.999
Total score	104	0.964	0.998

### 3.2. Validity of the internal structure

The Pearson’s coefficients for the OBS-SV domain scores ranged from 0.771 to 0.943, and they were all significantly, positively and strongly linked with one another (*p* < 0.01). The OBS overall score and each OBS-SV domain score showed strong correlation (see Table 3).

**Table 3:** Correlations among the OBS-SV domains.^a^

OBS spectrum domains	Doubt	Hypercontrol	Temporal dimension	Perfectionism	Repetition and automation	Obsessive themes
Doubt	–	–	–	–	–	–
Hypercontrol	0.722	–	–	–	–	–
Temporal dimension	0.659	0.691	–	–	–	
Perfectionism	0.510	0.705	0.529	–	–	–
Repetition and automation	0.565	0.671	0.615	0.614	–	–
Obsessive themes	0.595	0.687	0.622	0.539	0.590	–
Total score	0.813	0.943	0.784	0.787	0.771	0.833

a Pearson’s correlation coefficients were all significant at the *p* < 0.01 level, two tailed.

### 3.3. Convergent validity

Table 4 shows Pearson’s correlation coefficients for the correlation of OBS-SV total and domain scores with the Y-BOCS *global severity* item and total score. All the correlation coefficients appeared strong, statistically significant and positive.

**Table 4:** Correlations between the OBS-SV domains and Y-BOCS total score.^a^

OBS-SVdomains	Global Severity	Y-BOCS total score
Doubt	0.611	0.579
Hypercontrol	0.701	0.732
Temporal dimension	0.583	0.592
Perfectionism	0.654	0.693
Repetition and automation	0.644	0.689
Obsessive themes	0.680	0.743
Total score	0.750	0.817

a Pearson’s correlation coefficients were all significant at the *p* < 0.01 level, two tailed.

### 3.4. Known-groups validity

**Figure 1:**
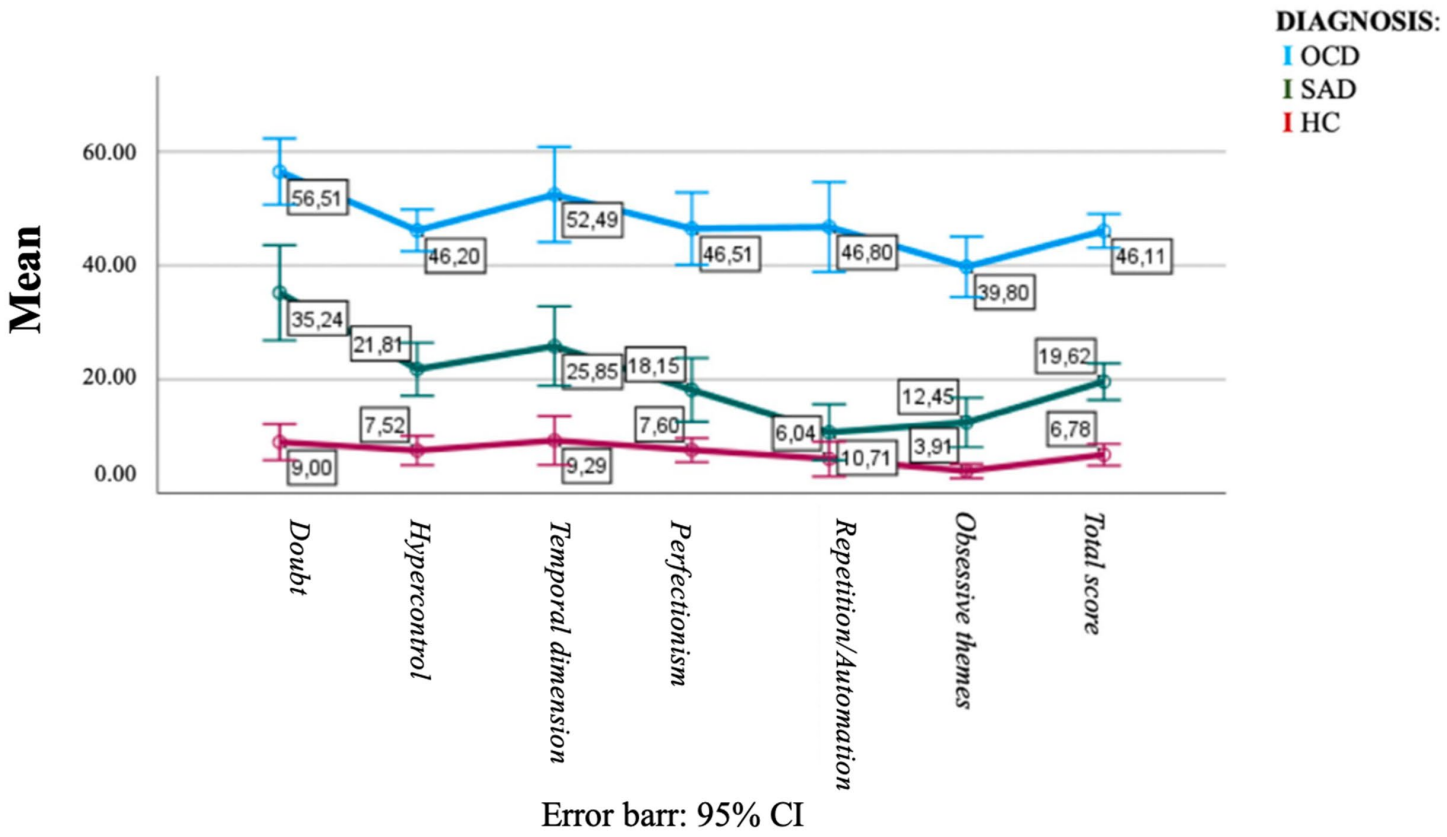
Trend of the OCD domain scores measured with the OBS- SV across groups.

**Figure 2:**
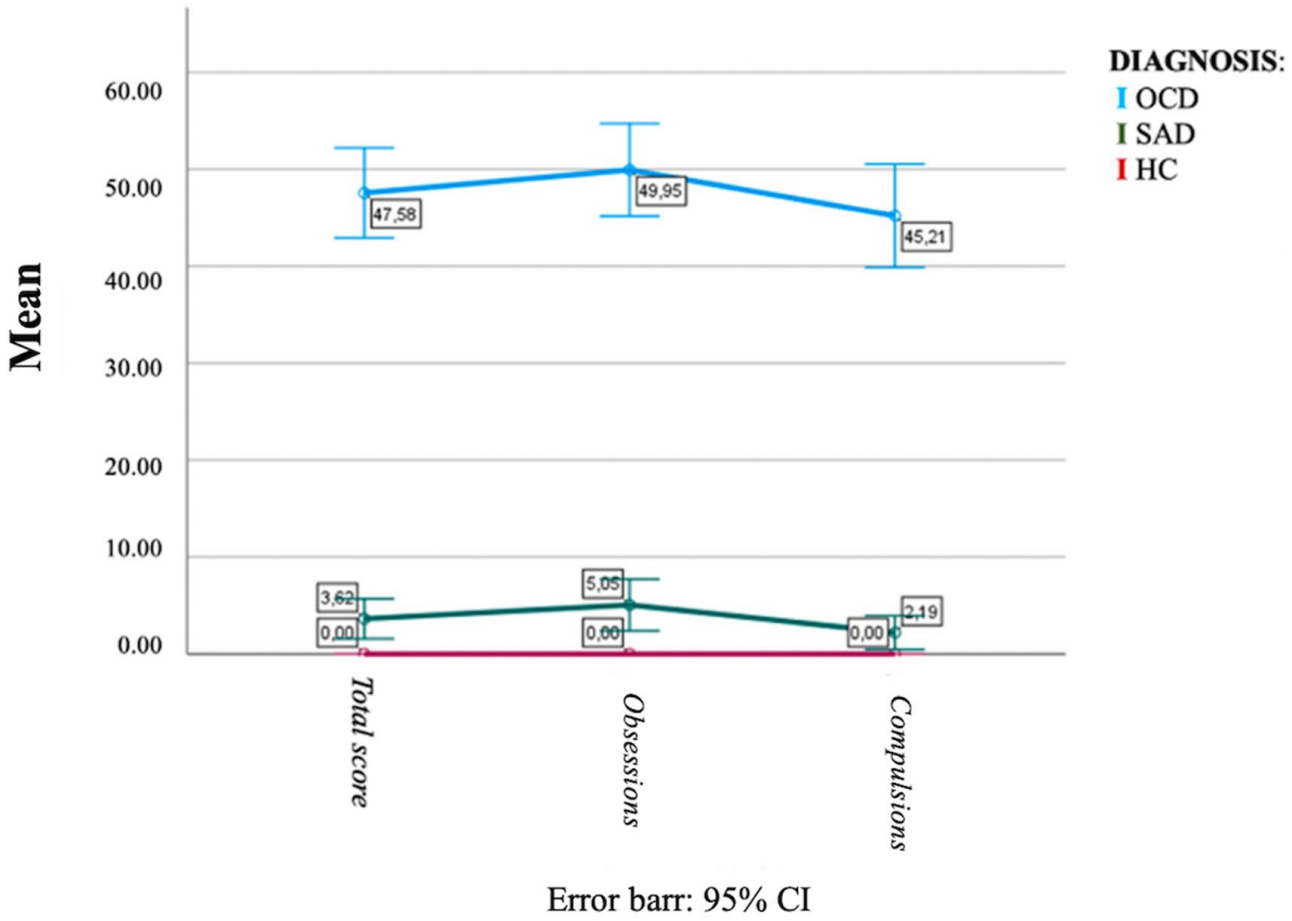
Trend of the OCD scores measured with the Y-BOCS across groups.

The ANOVA analysis revealed that there were significant differences between diagnostic groups for all OBS-SV domain and total scores (see Table 5). The OBS-SV total score was seen to rise dramatically and steadily from the HC, through the SAD, and up to the OC group, which reported the highest value. The OC group scored significantly higher in all domains than the SAD group, which in turn scored significantly higher than the HC group in all domains, with the exception of the *Repetition and automation* domain thus proving a good discriminant validity. Figure 1 illustrates the increasing trend of the OCD domain scores across groups. When comparing among groups the scores reported to the Y-BOCS, which is tailored to assess full-blown symptoms, OCD patients showed significantly higher scores than the other two groups. The SAD group reported higher scores than HCs but the difference was not statistically significant (see Figure 2).

**Table 5:** Comparison of OBS-SV total and domain scores among diagnostic groups.

OBS domains	HC (mean ± SD)	SF (mean ± SD)	OC (mean ± SD)	*F*	*p*	*Post-hoc* comparison^a^
Doubt	0.90 ± 1.23	3.52 ± 2.68	5.65 ± 1.89	76.86	< 0.001	HC < SP < OC
Hypercontrol	2.78 ± 3.69	8.07 ± 5.52	17.09 ± 4.41	127.21	< 0.001	HC < SP < OC
Temporal dimension	0.65 ± 1.16	1.81 ± 1.56	3.67 ± 1.90	49.13	< 0.001	HC < SP < OC
Perfectionism	1.22 ± 1.32	2.90 ± 2.88	7.44 ± 3.30	78.49	< 0.001	HC < SP < OC
Repetition and automation	0.48 ± 0.95	0.86 ± 1.26	3.74 ± 2.05	71.27	< 0.001	HC < OC, SP < OC
Obsessive themes	1.02 ± 1.27	3.24 ± 3.63	10.35 ± 4.49	108.64	< 0.001	HC < SP < OC
Total score	7.05 ± 7.67	20.40 ± 10.79	47.95 ± 10.02	87.82	<0.001	HC < SP < OC

^a^*p* < 0.05.

## 4. Discussion

The goal of this work was to introduce the OBS-SV, a clinical tool inspired by a dimensional approach to psychopathology, in light of the spectrum model (Cassano et al., 1997, 1998, 1999; Dell’Osso et al., 2003, 2016a,b, 2017, 2022). The OBS-SV investigates not only the prototypic symptoms of OCD but also unusual manifestations, temperamental traits, and other noteworthy clinical aspects linked to the main symptoms. For example, the OBS-SV allows to investigate the pervasive perfectionism (that although being a key factor in obsessive–compulsive personality disorder, is not necessarily present in OCD) thoughts of hurting someone else or themselves, moral absolutism, emotional control such as being unspontaneous, cold or detached toward others or being hypercritical, umorless or unimaginative. Results provided evidence of the validity and reliability of the OBS-SV administered to participants with a clinical diagnosis of OCD and SAD as well as HC. Using various dimensional measures of OCD, we discovered strong convergent validity, great internal consistency, and test–retest reliability. As expected, the questionnaire functioned differently among the three groups studied, with a progressive increase of the OBS-SV score going from healthy controls to SAD patients up to OCD participants.

The OBS–SV scores displayed positive, strong and significant correlations with the YBOCS, one of the most utilized instruments currently adopted to assess OC symptoms and features (Greeven et al., 2009; Anagnostou et al., 2011; Boyette et al., 2011; Ji et al., 2020). Compared to the previous version of the questionnaire that reported an internal consistency for all domains that ranged from moderate to substantial (0.60–0.90), the results from the new version confirmed its superiority showing all domains’ Cronbach alpha values above the value of 0.7 and an overall score of the scale (α = 0.964). Moreover, the OBS-SV exceeded the results reported from the previous scale both for the correlation between the domains (0.472–0.758 versus 0.771–0.943), and for the correlation with the alternative measure analyzed (0.58–9.82 versus 0.49–0.70).

Interestingly, the OBS–SV questionnaire seemed to be a useful tool for identifying subthreshold OC traits also in the healthy population and in SAD group, showing an increasing gradient of obsessive traits from the HC, passing through SAD subjects up to the OCD group. The different grades of OC spectrum among group seemed instead to be less clearly identified by the YBOCS, as highlighted by the comparison of the graphs of normalized OBS–SV and Y-BOCS scores among groups (Figures 1, 2). However, the presence of intermediate levels of OC traits in the SAD group is line with previous literature in the field, which frequently highlighted several OC traits among SAD patients, as well as overlapping features between the two disorders, and further support the spectrum model of psychopathology (Lochner and Stein, 2010; Egan et al., 2011; Dell’Osso et al., 2014, 2015; Marazziti et al., 2014; de Vries et al., 2018; Carpita et al., 2020).

Globally, our findings support the ability of the OBS-SV to properly detect the whole spectrum of OCD, from the subthreshold manifestations to the full-blown clinical picture. These results should be considered in light of some limitations. The principal limit of this work is the relatively limited sample size, which may, limit the extensibility of our data. Secondly, the OBS-SV, being a self-report questionnaire, may be at risk of underestimating or overestimating symptoms depending on the subjects’ judgment, and thus could be considered less precise when compared to the evaluation of the clinician. Additionally, we did not perform any inter-rater reliability tests nor the OBS-SV was administered to any pilot sample. Moreover, given the presence of a SAD group and the associations of SAD and OCD symptoms, it could have been interesting to examine the discriminant validity with a SAD measure. Likelywise, it would have also been interesting to assess the convergent validity of the OBS-SV with the OBS-SR, however, during the recruitment, the sample was only assessed with the OBS-SV and the Y-BOCS as an alternative measure of OCD.

The OBS-SV demonstrated good psychometric properties within the context of the aforementioned restrictions, and our findings offer a coherent construct of the OBS-SV with high internal consistency, strong test–retest reliability, and significant and positive convergent validity with alternative dimensional measures of OCD, such as the Y-BOCS. When compared with the previous version of the instrument and, the OBS-SV has the advantage of being more time efficient and more in line with the current conceptualization of OCD. Moreover, its use in the clinical setting, before the clinical interview could allow the clinician to have a general assessment of the symptomatologic profile of the subject and consent a faster and deeper investigation on some relevant symptomatologic domains. In this framework, it should be noted that, besides SAD, OC features have been also reported in a wide range of psychiatric conditions, ranging from neurodevelopmental disorders, to mood disorders, feeding and eating disorders and personality disorders, often worsening the clinical picture and response to treatments (McKay et al., 2000; Mukai et al., 2011; Postorino et al., 2017; Pazuniak and Pekrul, 2020; Tonna et al., 2021; Amianto et al., 2022; Reilly et al., 2022).

In conclusion, the OBS-SV not only demonstrated a significant convergent validity with other dimensional OCD measures, excellent internal consistency, and test–retest reliability but also, unlike the other commonly used measures, show the added value to be able to assess the wide spectrum of OCD manifestation, from the full-blown disorder to the subclinical manifestation, uncommon presentations, temperamental characteristics and other features related to the primary symptoms. Indeed, when evaluated with the Y-BOCS – a instrument designed to assess only full-blown manifestation – OCD patients scored significantly higher than the other two groups, but the latter had no significant differences with each other (Figure 2). On the contrary, the OBS-SV showed to have the specific value to be able to distinguish the presence of obsessive compulsive traits even in the SAD and HC groups, reporting a significant difference between them (Figure 1). The availability of an instrument able to detect sub-syndromic and atypical manifestations of this condition in both the general population and in clinical samples of patients with other mental disorders, may undoubtedly help in improving the diagnostic assessment and the treatment project of the patients, as well as support preventive and screening strategies in the general population.

## Data availability statement

The original contributions presented in the study are included in the article/Supplementary material, further inquiries can be directed to the corresponding author.

## Ethics statement

The studies involving human participants were reviewed and approved by Comitato Etico Regionale per la Sperimentazione Clinica della Regione Toscana. The patients/participants provided their written informed consent to participate in this study.

## Author contributions

LD’O conceived the work. All authors collected the data processed in the study. GM did statistical analysis. BN, CB, and LD’O drafted the manuscript. BC and LD’O revised the work. All authors provided approval of the version to be published.

## Conflict of interest

The authors declare that the research was conducted in the absence of any commercial or financial relationships that could be construed as a potential conflict of interest.

## Publisher’s note

All claims expressed in this article are solely those of the authors and do not necessarily represent those of their affiliated organizations, or those of the publisher, the editors and the reviewers. Any product that may be evaluated in this article, or claim that may be made by its manufacturer, is not guaranteed or endorsed by the publisher.
