# Supplementary material for: Validation of the short version of the obsessive compulsive spectrum questionnaire
Source: Front Psychol. 2023 Jun 27;14:1157636. doi: 10.3389/fpsyg.2023.1157636 (PMC10333544; doi:10.3389/fpsyg.2023.1157636)
Supplement: Supplementary file 1 [file Table_1.doc]

**Supplementary Table 1:** Domain descriptives in the total sample

| **Domains** | | | **Statistic** | **Std. Error** |
| --- | --- | --- | --- | --- |
| ***Doubt*** | **Mean** | | 3.069 | 0.231 |
|  | **95% C.I. for Mean** | Lower Bound | 2.613 |  |
|  |  | Upper Bound | 3.525 |  |
|  | **5% Trimmed Mean** | | 2.910 |  |
|  | **Median** | | 2.000 |  |
|  | **Variance** | | 7.717 |  |
|  | **Std. Deviation** | | 2.778 |  |
|  | **Minimum** | | 0.00 |  |
|  | **Maximum** | | 9.00 |  |
|  | **Range** | | 9.00 |  |
|  | **Interquartile Range** | | 4.00 |  |
|  | **Skewness** | | 0.637 | 0.201 |
|  | **Kurtosis** | | -0.637 | 0.400 |
| ***Hypercontrol*** | **Mean** | | 8.559 | 0.619 |
|  | **95% C.I. for Mean** | Lower Bound | 7.334 |  |
|  |  | Upper Bound | 9.783 |  |
|  | **5% Trimmed Mean** | | 8.162 |  |
|  | **Median** | | 6.000 |  |
|  | **Variance** | | 55.651 |  |
|  | **Std. Deviation** | | 7.460 |  |
|  | **Minimum** | | 0.00 |  |
|  | **Maximum** | | 27.00 |  |
|  | **Range** | | 27.00 |  |
|  | **Interquartile Range** | | 13.00 |  |
|  | **Skewness** | | 0.686 | 0.201 |
|  | **Kurtosis** | | -0.759 | 0.400 |
| ***Temporal dimension*** | **Mean** | | 1.883 | 0.164 |
|  | **95% C.I. for Mean** | Lower Bound | 1.559 |  |
|  |  | Upper Bound | 2.207 |  |
|  | **5% Trimmed Mean** | | 1.713 |  |
|  | **Median** | | 1.000 |  |
|  | **Variance** | | 3.896 |  |
|  | **Std. Deviation** | | 1.974 |  |
|  | **Minimum** | | 0.00 |  |
|  | **Maximum** | | 7.00 |  |
|  | **Range** | | 7.00 |  |
|  | **Interquartile Range** | | 3.00 |  |
|  | **Skewness** | | 0.924 | 0.201 |
|  | **Kurtosis** | | 0.030 | 0.400 |
| ***Perfectionism*** | **Mean** | | 3.552 | 0.301 |
|  | **95% C.I. for Mean** | Lower Bound | 2.956 |  |
|  |  | Upper Bound | 4.147 |  |
|  | **5% Trimmed Mean** | | 3.243 |  |
|  | **Median** | | 2.000 |  |
|  | **Variance** | | 13.166 |  |
|  | **Std. Deviation** | | 3.628 |  |
|  | **Minimum** | | 0.00 |  |
|  | **Maximum** | | 16.00 |  |
|  | **Range** | | 16.00 |  |
|  | **Interquartile Range** | | 5.00 |  |
|  | **Skewness** | | 1.146 | 0.201 |
|  | **Kurtosis** | | .803 | 0.400 |
| ***Repetition and automation*** | **Mean** | | 1.558 | 0.168 |
|  | **95% C.I. for Mean** | Lower Bound | 1.226 |  |
|  |  | Upper Bound | 1.891 |  |
|  | **5% Trimmed Mean** | | 1.345 |  |
|  | **Median** | | 1.000 |  |
|  | **Variance** | | 4.095 |  |
|  | **Std. Deviation** | | 2.024 |  |
|  | **Minimum** | | 0.00 |  |
|  | **Maximum** | | 8.00 |  |
|  | **Range** | | 8.00 |  |
|  | **Interquartile Range** | | 3.00 |  |
|  | **Skewness** | | 1.333 | 0.201 |
|  | **Kurtosis** | | 1.021 | 0.400 |
| ***Obsessive themes*** | **Mean** | | 4.428 | 0.423 |
|  | **95% C.I. for Mean** | Lower Bound | 3.590 |  |
|  |  | Upper Bound | 5.265 |  |
|  | **5% Trimmed Mean** | | 4.010 |  |
|  | **Median** | | 2.000 |  |
|  | **Variance** | | 26.010 |  |
|  | **Std. Deviation** | | 5.100 |  |
|  | **Minimum** | | 0.00 |  |
|  | **Maximum** | | 18.00 |  |
|  | **Range** | | 18.00 |  |
|  | **Interquartile Range** | | 8.00 |  |
|  | **Skewness** | | 1.050 | 0.201 |
|  | **Kurtosis** | | -0.120 | 0.400 |
| **Total score** | **Mean** | | 23.048 | 1.620 |
|  | **95% C.I. for Mean** | Lower Bound | 19.846 |  |
|  |  | Upper Bound | 26.250 |  |
|  | **5% Trimmed Mean** | | 22.163 |  |
|  | **Median** | | 17.000 |  |
|  | **Variance** | | 380.505 |  |
|  | **Std. Deviation** | | 19.506 |  |
|  | **Minimum** | | 0.00 |  |
|  | **Maximum** | | 67.00 |  |
|  | **Range** | | 67.00 |  |
|  | **Interquartile Range** | | 35.50 |  |
|  | **Skewness** | | 0.613 | 0.201 |
|  | **Kurtosis** | | -0.987 | 0.400 |

**Supplementary Table 1:** Domain descriptives divided by diagnosis

|  | **Diagnosis** | | | **Statistic** | **Std. Error** |
| --- | --- | --- | --- | --- | --- |
| **Doubt** | DOC | **Mean** | | 5.651 | 0.288 |
|  |  | **95% C.I. for Mean** | Lower Bound | 5.070 |  |
|  |  |  | Upper Bound | 6.232 |  |
|  |  | **5% Trimmed Mean** | | 5.612 |  |
|  |  | **Median** | | 6.000 |  |
|  |  | **Variance** | | 3.566 |  |
|  |  | **Std. Deviation** | | 1.888 |  |
|  |  | **Minimum** | | 3.00 |  |
|  |  | **Maximum** | | 9.00 |  |
|  |  | **Range** | | 6.00 |  |
|  |  | **Interquartile Range** | | 3.00 |  |
|  |  | **Skewness** | | 0.510 | 0.361 |
|  |  | **Kurtosis** | | - 0.615 | 0.709 |
|  | **SAD** | **Mean** | | 3.524 | 0.413 |
|  |  | **95% C.I. for Mean** | Lower Bound | 2.689 |  |
|  |  |  | Upper Bound | 4.359 |  |
|  |  | **5% Trimmed Mean** | | 3.415 |  |
|  |  | **Median** | | 3.000 |  |
|  |  | **Variance** | | 7.182 |  |
|  |  | **Std. Deviation** | | 2.680 |  |
|  |  | **Minimum** | | 0.00 |  |
|  |  | **Maximum** | | 9.00 |  |
|  |  | **Range** | | 9.00 |  |
|  |  | **Interquartile Range** | | 3.00 |  |
|  |  | **Skewness** | | 0.675 | 0.365 |
|  |  | **Kurtosis** | | - 0.426 | 0.717 |
|  | **HC** | **Mean** | | 0.900 | 0.159 |
|  |  | **95% C.I.for Mean** | Lower Bound | 0.582 |  |
|  |  |  | Upper Bound | 1.218 |  |
|  |  | **5% Trimmed Mean** | | 0.740 |  |
|  |  | **Median** | | 0.500 |  |
|  |  | **Variance** | | 1.515 |  |
|  |  | **Std. Deviation** | | 1.231 |  |
|  |  | **Minimum** | | 9.00 |  |
|  |  | **Maximum** | | 5.00 |  |
|  |  | **Range** | | 5.00 |  |
|  |  | **Interquartile Range** | | 1.00 |  |
|  |  | **Skewness** | | 1.719 | 0.309 |
|  |  | **Kurtosis** | | 2.936 | 0.608 |
| ***Hypercontrol*** | **OC** | **Mean** | | 17.093 | 0.673 |
|  |  | **95% C.I. for Mean** | Lower Bound | 15.735 |  |
|  |  |  | Upper Bound | 18.451 |  |
|  |  | **5% Trimmed Mean** | | 17.155 |  |
|  |  | **Median** | | 17.000 |  |
|  |  | **Variance** | | 19.467 |  |
|  |  | **Std. Deviation** | | 4.412 |  |
|  |  | **Minimum** | | 6.00 |  |
|  |  | **Maximum** | | 26.00 |  |
|  |  | **Range** | | 20.00 |  |
|  |  | **Interquartile Range** | | 7.00 |  |
|  |  | **Skewness** | | - 0.119 | 0.361 |
|  |  | **Kurtosis** | | - 0.485 | 0.709 |
|  | **SF** | **Mean** | | 8.071 | 0.852 |
|  |  | **95% C.I. for Mean** | Lower Bound | 6.350 |  |
|  |  |  | Upper Bound | 9.793 |  |
|  |  | **5% Trimmed Mean** | | 7.479 |  |
|  |  | **Median** | | 6.500 |  |
|  |  | **Variance** | | 30.507 |  |
|  |  | **Std. Deviation** | | 5.523 |  |
|  |  | **Minimum** | | 0.00 |  |
|  |  | **Maximum** | | 27.00 |  |
|  |  | **Range** | | 27.00 |  |
|  |  | **Interquartile Range** | | 4.00 |  |
|  |  | **Skewness** | | 1.959 | 0.365 |
|  |  | **Kurtosis** | | 4.284 | 0.717 |
|  | **HC** | **Mean** | | 2.783 | 0.476 |
|  |  | **95% C.I. for Mean** | Lower Bound | 1.831 |  |
|  |  |  | Upper Bound | 3.736 |  |
|  |  | **5% Trimmed Mean** | | 2.222 |  |
|  |  | **Median** | | 2.000 |  |
|  |  | **Variance** | | 13.596 |  |
|  |  | **Std. Deviation** | | 3.687 |  |
|  |  | **Minimum** | | 0.00 |  |
|  |  | **Maximum** | | 16.00 |  |
|  |  | **Range** | | 16.00 |  |
|  |  | **Interquartile Range** | | 2.00 |  |
|  |  | **Skewness** | | 2.652 | 0.309 |
|  |  | **Kurtosis** | | 7.011 | 0.608 |
| ***Temporal dimension*** | **OC** | **Mean** | | 3.674 | 0.289 |
|  |  | **95% C.I. for Mean** | Lower Bound | 3.090 |  |
|  |  |  | Upper Bound | 4.259 |  |
|  |  | **5% Trimmed Mean** | | 3.664 |  |
|  |  | **Median** | | 4.000 |  |
|  |  | **Variance** | | 3.606 |  |
|  |  | **Std. Deviation** | | 1.899 |  |
|  |  | **Minimum** | | 0.00 |  |
|  |  | **Maximum** | | 7.00 |  |
|  |  | **Range** | | 7.00 |  |
|  |  | **Interquartile Range** | | 3.00 |  |
|  |  | **Skewness** | | 0.166 | 0.361 |
|  |  | **Kurtosis** | | - 0.720 | 0.709 |
|  | **SF** | **Mean** | | 1.809 | 0.241 |
|  |  | **95% C.I. for Mean** | Lower Bound | 1.322 |  |
|  |  |  | Upper Bound | 2.297 |  |
|  |  | **5% Trimmed Mean** | | 1.709 |  |
|  |  | **Median** | | 2.000 |  |
|  |  | **Variance** | | 2.451 |  |
|  |  | **Std. Deviation** | | 1.565 |  |
|  |  | **Minimum** | | 0.00 |  |
|  |  | **Maximum** | | 7.00 |  |
|  |  | **Range** | | 7.00 |  |
|  |  | **Interquartile Range** | | 3.00 |  |
|  |  | **Skewness** | | 0.853 | 0.365 |
|  |  | **Kurtosis** | | 1.269 | 0.717 |
|  | **HC** | **Mean** | | 0.650 | 0.150 |
|  |  | **95% C.I.for Mean** | Lower Bound | 0.350 |  |
|  |  |  | Upper Bound | 0.950 |  |
|  |  | **5% Trimmed Mean** | | 0.481 |  |
|  |  | **Median** | | 0.000 |  |
|  |  | **Variance** | | 1.350 |  |
|  |  | **Std. Deviation** | | 1.162 |  |
|  |  | **Minimum** | | 0.00 |  |
|  |  | **Maximum** | | 5.00 |  |
|  |  | **Range** | | 5.00 |  |
|  |  | **Interquartile Range** | | 1.00 |  |
|  |  | **Skewness** | | 2.271 | 0.309 |
|  |  | **Kurtosis** | | 5.367 | 0.608 |
| ***Perfectionism*** | **OC** | **Mean** | | 7.442 | 0.504 |
|  |  | **95% C.I. for Mean** | Lower Bound | 6.425 |  |
|  |  |  | Upper Bound | 8.459 |  |
|  |  | **5% Trimmed Mean** | | 7.499 |  |
|  |  | **Median** | | 8.000 |  |
|  |  | **Variance** | | 10.919 |  |
|  |  | **Std. Deviation** | | 3.304 |  |
|  |  | **Minimum** | | 0.00 |  |
|  |  | **Maximum** | | 16.00 |  |
|  |  | **Range** | | 16.00 |  |
|  |  | **Interquartile Range** | | 4.00 |  |
|  |  | **Skewness** | | - 0.319 | 0.361 |
|  |  | **Kurtosis** | | 0.853 | 0.709 |
|  | **SF** | **Mean** | | 2.905 | 0.444 |
|  |  | **95% C.I for Mean** | Lower Bound | 2.008 |  |
|  |  |  | Upper Bound | 3.802 |  |
|  |  | **5% Trimmed Mean** | | 2.574 |  |
|  |  | **Median** | | 3.000 |  |
|  |  | **Variance** | | 8.283 |  |
|  |  | **Std. Deviation** | | 2.878 |  |
|  |  | **Minimum** | | 0.00 |  |
|  |  | **Maximum** | | 16.00 |  |
|  |  | **Range** | | 16.00 |  |
|  |  | **Interquartile Range** | | 3.00 |  |
|  |  | **Skewness** | | 2.440 | 0.365 |
|  |  | **Kurtosis** | | 9.636 | 0.717 |
|  | **HC** | **Mean** | | 1.217 | 0.170 |
|  |  | **95% C.I. for Mean** | Lower Bound | 0.877 |  |
|  |  |  | Upper Bound | 1.557 |  |
|  |  | **5% Trimmed Mean** | | 1.074 |  |
|  |  | **Median** | | 1.000 |  |
|  |  | **Variance** | | 1.732 |  |
|  |  | **Std. Deviation** | | 1.316 |  |
|  |  | **Minimum** | | 0.00 |  |
|  |  | **Maximum** | | 7.00 |  |
|  |  | **Range** | | 7.00 |  |
|  |  | **Interquartile Range** | | 2.00 |  |
|  |  | **Skewness** | | 1.800 | 0.309 |
|  |  | **Kurtosis** | | 5.207 | 0.608 |
| ***Repetition*** | **OC** | **Mean** | | 3.744 | 0.312 |
|  |  | **95% C.I. for Mean** | Lower Bound | 3.114 |  |
|  |  |  | Upper Bound | 4.374 |  |
|  |  | **5% Trimmed Mean** | | 3.720 |  |
|  |  | **Median** | | 4.000 |  |
|  |  | **Variance** | | 4.195 |  |
|  |  | **Std. Deviation** | | 2.048 |  |
|  |  | **Minimum** | | 0.00 |  |
|  |  | **Maximum** | | 8.00 |  |
|  |  | **Range** | | 8.00 |  |
|  |  | **Interquartile Range** | | 2.00 |  |
|  |  | **Skewness** | | 0.189 | 0.361 |
|  |  | **Kurtosis** | | - 0.255 | 0.709 |
|  | **SF** | **Mean** | | 0.857 | 0.194 |
|  |  | **95% C.I. for Mean** | Lower Bound | 0.464 |  |
|  |  |  | Upper Bound | 1.250 |  |
|  |  | **5% Trimmed Mean** | | 0.682 |  |
|  |  | **Median** | | 0.000 |  |
|  |  | **Variance** | | 1.589 |  |
|  |  | **Std. Deviation** | | 1.260 |  |
|  |  | **Minimum** | | 0.00 |  |
|  |  | **Maximum** | | 6.00 |  |
|  |  | **Range** | | 6.00 |  |
|  |  | **Interquartile Range** | | 2.00 |  |
|  |  | **Skewness** | | 2.123 | 0.365 |
|  |  | **Kurtosis** | | 5.994 | 0.717 |
|  | **HC** | **Mean** | | 0.483 | 0.122 |
|  |  | **95% C.I. for Mean** | Lower Bound | 0.238 |  |
|  |  |  | Upper Bound | 0.728 |  |
|  |  | **5% Trimmed Mean** | | 0.333 |  |
|  |  | **Median** | | 0.000 |  |
|  |  | **Variance** | | 0.898 |  |
|  |  | **Std. Deviation** | | 0.948 |  |
|  |  | **Minimum** | | 0.00 |  |
|  |  | **Maximum** | | 5.00 |  |
|  |  | **Range** | | 5.00 |  |
|  |  | **Interquartile Range** | | 1.00 |  |
|  |  | **Skewness** | | 2.707 | 0.309 |
|  |  | **Kurtosis** | | 8.834 | 0.608 |
| ***Obsessive themes*** | **OC** | **Mean** | | 10.348 | 0.684 |
|  |  | **95% C.I. for Mean** | Lower Bound | 8.968 |  |
|  |  |  | Upper Bound | 11.729 |  |
|  |  | **5% Trimmed Mean** | | 10.517 |  |
|  |  | **Median** | | 10.000 |  |
|  |  | **Variance** | | 20.137 |  |
|  |  | **Std. Deviation** | | 4.487 |  |
|  |  | **Minimum** | | 0.00 |  |
|  |  | **Maximum** | | 18.00 |  |
|  |  | **Range** | | 18.00 |  |
|  |  | **Interquartile Range** | | 6.00 |  |
|  |  | **Skewness** | | - 0.466 | 0.361 |
|  |  | **Kurtosis** | | - 0.200 | 0.709 |
|  | **SF** | **Mean** | | 3.238 | 0.560 |
|  |  | **95% C.I. for Mean** | Lower Bound | 2.108 |  |
|  |  |  | Upper Bound | 4.369 |  |
|  |  | **5% Trimmed Mean** | | 2.886 |  |
|  |  | **Median** | | 2.000 |  |
|  |  | **Variance** | | 13.161 |  |
|  |  | **Std. Deviation** | | 3.628 |  |
|  |  | **Minimum** | | 0.00 |  |
|  |  | **Maximum** | | 16.00 |  |
|  |  | **Range** | | 16.00 |  |
|  |  | **Interquartile Range** | | 4.50 |  |
|  |  | **Skewness** | | 1.469 | 0.365 |
|  |  | **Kurtosis** | | 2.380 | 0.717 |
|  | **HC** | **Mean** | | 1.017 | 0.164 |
|  |  | **95% C.I. for Mean** | Lower Bound | 0.689 |  |
|  |  |  | Upper Bound | 1.344 |  |
|  |  | **5% Trimmed Mean** | | 0.907 |  |
|  |  | **Median** | | 0.500 |  |
|  |  | **Variance** | | 1.610 |  |
|  |  | **Std. Deviation** | | 1.269 |  |
|  |  | **Minimum** | | 0.00 |  |
|  |  | **Maximum** | | 5.00 |  |
|  |  | **Range** | | 5.00 |  |
|  |  | **Interquartile Range** | | 2.00 |  |
|  |  | **Skewness** | | 1.101 | 0.309 |
|  |  | **Kurtosis** | | 0.420 | 0.608 |
| **Total score** | **OC** | **Mean** | | 47.953 | 1.529 |
|  |  | **95% C.I. for Mean** | Lower Bound | 44.867 |  |
|  |  |  | Upper Bound | 51.040 |  |
|  |  | **5% Trimmed Mean** | | 48.141 |  |
|  |  | **Median** | | 49.000 |  |
|  |  | **Variance** | | 100.569 |  |
|  |  | **Std. Deviation** | | 10.028 |  |
|  |  | **Minimum** | | 27.00 |  |
|  |  | **Maximum** | | 67.00 |  |
|  |  | **Range** | | 40.00 |  |
|  |  | **Interquartile Range** | | 13.00 |  |
|  |  | **Skewness** | | - 0.312 | 0.361 |
|  |  | **Kurtosis** | | - 0.592 | 0.709 |
|  | **SF** | **Mean** | | 20.405 | 1.666 |
|  |  | **95% C.I. for Mean** | Lower Bound | 17.041 |  |
|  |  |  | Upper Bound | 23.769 |  |
|  |  | **5% Trimmed Mean** | | 19.632 |  |
|  |  | **Median** | | 18.000 |  |
|  |  | **Variance** | | 116.539 |  |
|  |  | **Std. Deviation** | | 10.795 |  |
|  |  | **Minimum** | | 1.00 |  |
|  |  | **Maximum** | | 57.00 |  |
|  |  | **Range** | | 56.00 |  |
|  |  | **Interquartile Range** | | 10.50 |  |
|  |  | **Skewness** | | 1.387 | 0.365 |
|  |  | **Kurtosis** | | 3.200 | 0.717 |
|  | **HC** | **Mean** | | 7.050 | 0.990 |
|  |  | **95% C.I. for Mean** | Lower Bound | 5.069 |  |
|  |  |  | Upper Bound | 9.031 |  |
|  |  | **5% Trimmed Mean** | | 6.018 |  |
|  |  | **Median** | | 5.000 |  |
|  |  | **Variance** | | 58.794 |  |
|  |  | **Std. Deviation** | | 7.668 |  |
|  |  | **Minimum** | | 0.00 |  |
|  |  | **Maximum** | | 35.00 |  |
|  |  | **Range** | | 35.00 |  |
|  |  | **Interquartile Range** | | 5.00 |  |
|  |  | **Skewness** | | 2.390 | 0.309 |
|  |  | **Kurtosis** | | 5.797 | 0.608 |

**Supplementary Table 3:**  Items response percentage by diagnosis

|  | | | **DIAGNOSIS** | | | **Total** |
| --- | --- | --- | --- | --- | --- | --- |
|  |  |  | **DOC** | **SAD** | **HC** |  |
| **OBS-SV1** | **Negative response** | **Count** | 17_a_ | 32_b_ | 60_c_ | 109 |
|  |  | **% within diagnosis** | 39.5% | 76.2% | 100.0% | 75.2% |
|  | **Positive**  **response** | **Count** | 26_a_ | 10_b_ | 0_c_ | 36 |
|  |  | **% within diagnosis** | 6.5% | 23.8% | 0,0% | 24.8% |
| ***Pearson Chi-Square*** | | | *49.101* | | | |
| ***Likelihood Ratio*** | | | *58.707* | | | |
| ***Linear-by-Linear Association*** | | | *48.113* | | | |
| **OBS-SV2** | **Negative response** | **Count** | 18_a_ | 25_a_ | 53_b_ | 96 |
|  |  | **% within diagnosis** | 41.9% | 59.5% | 88.3% | 66.2% |
|  | **Positive**  **response** | **Count** | 25_a_ | 17_a_ | 7_b_ | 49 |
|  |  | **% within diagnosis** | 58.1% | 40.5% | 11.7% | 33.8% |
| ***Pearson Chi-Square*** | | | *25.360* | | | |
| ***Likelihood Ratio*** | | | *27.115* | | | |
| ***Linear-by-Linear Association*** | | | *24.777* | | | |
| **OBS-SV3** | **Negative response** | **Count** | 14_a_ | 27_b_ | 51_c_ | 92 |
|  |  | **% within diagnosis** | 32.6% | 64.3% | 85.0% | 63.4% |
|  | **Positive**  **response** | **Count** | 29_a_ | 15_b_ | 9_c_ | 53 |
|  |  | **% within diagnosis** | 67.4% | 35.7% | 15.0% | 36.6% |
| ***Pearson Chi-Square*** | | | *29.722* | | | |
| ***Likelihood Ratio*** | | | *30.654* | | | |
| ***Linear-by-Linear Association*** | | | *29.132* | | | |
| **OBS-SV4** | **Negative response** | **Count** | 15_a_ | 28_b_ | 53_c_ | 96 |
|  |  | **% within diagnosis** | 34.9% | 66.7% | 88.3% | 66.2% |
|  | **Positive**  **response** | **Count** | 28_a_ | 14_b_ | 7_c_ | 49 |
|  |  | **% within diagnosis** | 65.1% | 33.3% | 11.7% | 33.8% |
| ***Pearson Chi-Square*** | | | *31.990* | | | |
| ***Likelihood Ratio*** | | | *33.186* | | | |
| ***Linear-by-Linear Association*** | | | *31.433* | | | |
| **OBS-SV5** | **Negative response** | **Count** | 16_a_ | 18_a_ | 58_b_ | 92 |
|  |  | **% within diagnosis** | 37.2% | 42.9% | 96.7% | 63.4% |
|  | **Positive**  **response** | **Count** | 27_a_ | 24_a_ | 2_b_ | 53 |
|  |  | **% within diagnosis** | 62.8% | 57.1% | 3.3% | 36.6% |
| ***Pearson Chi-Square*** | | | *48.992* | | | |
| ***Likelihood Ratio*** | | | *58.726* | | | |
| ***Linear-by-Linear Association*** | | | *41.306* | | | |
| **OBS-SV6** | **Negative response** | **Count** | 17_a_ | 23_a_ | 53_b_ | 93 |
|  |  | **% within diagnosis** | 39.5% | 54.8% | 88.3% | 64.1% |
|  | **Positive**  **response** | **Count** | 26_a_ | 19_a_ | 7_b_ | 52 |
|  |  | **% within diagnosis** | 60.5% | 45.2% | 11.7% | 35.9% |
| ***Pearson Chi-Square*** | | | *28.192* | | | |
| ***Likelihood Ratio*** | | | *30.477* | | | |
| ***Linear-by-Linear Association*** | | | *26.923* | | | |
| **OBS-SV7** | **Negative response** | **Count** | 20_a_ | 28_a, b_ | 50_b_ | 98 |
|  |  | **% within diagnosis** | 46.5% | 66.7% | 83.3% | 67.6% |
|  | **Positive**  **response** | **Count** | 23_a_ | 14_a, b_ | 10_b_ | 47 |
|  |  | **% within diagnosis** | 53.5% | 33.3% | 16.7% | 32.4% |
| ***Pearson Chi-Square*** | | | *15.525* | | | |
| ***Likelihood Ratio*** | | | *15.750* | | | |
| ***Linear-by-Linear Association*** | | | *15.377* | | | |
| **OBS-SV8** | **Negative response** | **Count** | 16_a_ | 22_a_ | 59_b_ | 97 |
|  |  | **% within diagnosis** | 37.2% | 52.4% | 98.3% | 66.9% |
|  | **Positive**  **response** | **Count** | 27_a_ | 20_a_ | 1_b_ | 48 |
|  |  | **% within diagnosis** | 62.8% | 47.6% | 1.7% | 33.1% |
| ***Pearson Chi-Square*** | | | *47.886* | | | |
| ***Likelihood Ratio*** | | | *59.057* | | | |
| ***Linear-by-Linear Association*** | | | *44.412* | | | |
| **OBS-SV9** | **Negative response** | **Count** | 18_a_ | 25_a_ | 54_b_ | 97 |
|  |  | **% within diagnosis** | 41.9% | 59.5% | 90.0% | 66.9% |
|  | **Positive**  **response** | **Count** | 25_a_ | 17_a_ | 6_b_ | 48 |
|  |  | **% within diagnosis** | 58.1% | 40.5% | 10.0% | 33.1% |
| ***Pearson Chi-Square*** | | | *27.664* | | | |
| ***Likelihood Ratio*** | | | *29.956* | | | |
| ***Linear-by-Linear Association*** | | | *26.928* | | | |
| **OBS-SV10** | **Negative response** | **Count** | 9_a_ | 24_b_ | 54_c_ | 87 |
|  |  | **% within diagnosis** | 20.9% | 57.1% | 90.0% | 60.0% |
|  | **Positive**  **response** | **Count** | 34_a_ | 18_b_ | 6_c_ | 58 |
|  |  | **% within diagnosis** | 79.1% | 42.9% | 10.0% | 40.0% |
| ***Pearson Chi-Square*** | | | *49.992* | | | |
| ***Likelihood Ratio*** | | | *54.678* | | | |
| ***Linear-by-Linear Association*** | | | *49.612* | | | |
| **OBS-SV11** | **Negative response** | **Count** | 13_a_ | 27_b_ | 55_c_ | 95 |
|  |  | **% within diagnosis** | 30.2% | 64.3% | 91.7% | 65.5% |
|  | **Positive**  **response** | **Count** | 30_a_ | 15_b_ | 5_c_ | 50 |
|  |  | **% within diagnosis** | 69.8% | 35.7% | 8.3% | 34.5% |
| ***Pearson Chi-Square*** | | | *41.885* | | | |
| ***Likelihood Ratio*** | | | *44.943* | | | |
| ***Linear-by-Linear Association*** | | | *41.451* | | | |
| **OBS-SV12** | **Negative response** | **Count** | 20_a_ | 28_a_ | 58_b_ | 106 |
|  |  | **% within diagnosis** | 46.5% | 66.7% | 96.7% | 73.1% |
|  | **Positive**  **response** | **Count** | 23_a_ | 14_a_ | 2_b_ | 39 |
|  |  | **% within diagnosis** | 53.5% | 33.3% | 3.3% | 26.9% |
| ***Pearson Chi-Square*** | | | *33.292* | | | |
| ***Likelihood Ratio*** | | | *38.440* | | | |
| ***Linear-by-Linear Association*** | | | *32.700* | | | |
| **OBS-SV13** | **Negative response** | **Count** | 22_a_ | 33_b_ | 60_c_ | 115 |
|  |  | **% within diagnosis** | 51.2% | 78.6% | 100.0% | 79.3% |
|  | **Positive**  **response** | **Count** | 21_a_ | 9_b_ | 0_c_ | 30 |
|  |  | **% within diagnosis** | 48.8% | 21.4% | 0.0% | 20.7% |
| ***Pearson Chi-Square*** | | | *36.428* | | | |
| ***Likelihood Ratio*** | | | *44.614* | | | |
| ***Linear-by-Linear Association*** | | | *36.017* | | | |
| **OBS-SV14** | **Negative response** | **Count** | 9_a_ | 30_b_ | 53_b_ | 92 |
|  |  | **% within diagnosis** | 20.9% | 71.4% | 88.3% | 63.4% |
|  | **Positive**  **response** | **Count** | 34_a_ | 12_b_ | 7_b_ | 53 |
|  |  | **% within diagnosis** | 79.1% | 28.6% | 11.7% | 36.6% |
| ***Pearson Chi-Square*** | | | *50.693* | | | |
| ***Likelihood Ratio*** | | | *52.790* | | | |
| ***Linear-by-Linear Association*** | | | *46.768* | | | |
| **OBS-SV15** | **Negative response** | **Count** | 23_a_ | 38_b_ | 55_b_ | 116 |
|  |  | **% within diagnosis** | 53.5% | 90.5% | 91.7% | 80.0% |
|  | **Positive**  **response** | **Count** | 20_a_ | 4_b_ | 5_b_ | 29 |
|  |  | **% within diagnosis** | 46.5% | 9.5% | 8.3% | 20.0% |
| ***Pearson Chi-Square*** | | | *26.875* | | | |
| ***Likelihood Ratio*** | | | *24.878* | | | |
| ***Linear-by-Linear Association*** | | | *20.805* | | | |
| **OBS-SV16** | **Negative response** | **Count** | 21_a_ | 31_a, b_ | 48_b_ | 100 |
|  |  | **% within diagnosis** | 48.8% | 73.8% | 80.0% | 69.0% |
|  | **Positive**  **response** | **Count** | 22_a_ | 11_a, b_ | 12_b_ | 45 |
|  |  | **% within diagnosis** | 51.2% | 26.2% | 20.0% | 31.0% |
| ***Pearson Chi-Square*** | | | *12.013* | | | |
| ***Likelihood Ratio*** | | | *11.680* | | | |
| ***Linear-by-Linear Association*** | | | *10.720* | | | |
| **OBS-SV17** | **Negative response** | **Count** | 28_a_ | 30_a_ | 55_b_ | 113 |
|  |  | **% within diagnosis** | 65.1% | 71.4% | 91.7% | 77.9% |
|  | **Positive**  **response** | **Count** | 15_a_ | 12_a_ | 5_b_ | 32 |
|  |  | **% within diagnosis** | 34.9% | 28.6% | 8.3% | 22.1% |
| ***Pearson Chi-Square*** | | | *11.720* | | | |
| ***Likelihood Ratio*** | | | *12.763* | | | |
| ***Linear-by-Linear Association*** | | | *10.811* | | | |
| **OBS-SV18** | **Negative response** | **Count** | 16_a_ | 23_a_ | 56_b_ | 95 |
|  |  | **% within diagnosis** | 37.2% | 54.8% | 93.3% | 65.5% |
|  | **Positive**  **response** | **Count** | 27_a_ | 19_a_ | 4_b_ | 50 |
|  |  | **% within diagnosis** | 62.8% | 45.2% | 6.7% | 34.5% |
| ***Pearson Chi-Square*** | | | *37.951* | | | |
| ***Likelihood Ratio*** | | | *42.814* | | | |
| ***Linear-by-Linear Association*** | | | *36.253* | | | |
| **OBS-SV19** | **Negative response** | **Count** | 34_a_ | 32_a_ | 59_b_ | 125 |
|  |  | **% within diagnosis** | 79.1% | 76.2% | 98.3% | 86.2% |
|  | **Positive**  **response** | **Count** | 9_a_ | 10_a_ | 1_b_ | 20 |
|  |  | **% within diagnosis** | 20.9% | 23.8% | 1.7% | 13.8% |
| ***Pearson Chi-Square*** | | | *12.806* | | | |
| ***Likelihood Ratio*** | | | *15.947* | | | |
| ***Linear-by-Linear Association*** | | | *8.849* | | | |
| **OBS-SV20** | **Negative response** | **Count** | 32_a_ | 35_a_ | 60_b_ | 127 |
|  |  | **% within diagnosis** | 74.4% | 83.3% | 100.0% | 87.6% |
|  | **Positive**  **response** | **Count** | 11_a_ | 7_a_ | 0_b_ | 18 |
|  |  | **% within diagnosis** | 25.6% | 16.7% | 0,0% | 12.4% |
| ***Pearson Chi-Square*** | | | *16.060* | | | |
| ***Likelihood Ratio*** | | | *22.026* | | | |
| ***Linear-by-Linear Association*** | | | *15.543* | | | |
| **OBS-SV21** | **Negative response** | **Count** | 17_a_ | 30_b_ | 54_c_ | 101 |
|  |  | **% within diagnosis** | 39.5% | 71.4% | 90.0% | 69.7% |
|  | **Positive**  **response** | **Count** | 26_a_ | 12_b_ | 6_c_ | 44 |
|  |  | **% within diagnosis** | 60.5% | 28.6% | 10.0% | 30.3% |
| ***Pearson Chi-Square*** | | | *30.269* | | | |
| ***Likelihood Ratio*** | | | *31.012* | | | |
| ***Linear-by-Linear Association*** | | | *29.443* | | | |
| **OBS-SV22** | **Negative response** | **Count** | 23_a_ | 36_b_ | 56_b_ | 115 |
|  |  | **% within diagnosis** | 53.5% | 85.7% | 93.3% | 79.3% |
|  | **Positive**  **response** | **Count** | 20_a_ | 6_b_ | 4_b_ | 30 |
|  |  | **% within diagnosis** | 46.5% | 14.3% | 6.7% | 20.7% |
| ***Pearson Chi-Square*** | | | *25.713* | | | |
| ***Likelihood Ratio*** | | | *24.604* | | | |
| ***Linear-by-Linear Association*** | | | *22.824* | | | |
| **OBS-SV23** | **Negative response** | **Count** | 21_a_ | 29_a_ | 58_b_ | 108 |
|  |  | **% within diagnosis** | 48.8% | 69.0% | 96.7% | 74.5% |
|  | **Positive**  **response** | **Count** | 22_a_ | 13_a_ | 2_b_ | 37 |
|  |  | **% within diagnosis** | 51.2% | 31.0% | 3.3% | 25.5% |
| ***Pearson Chi-Square*** | | | *31.069* | | | |
| ***Likelihood Ratio*** | | | *35.607* | | | |
| ***Linear-by-Linear Association*** | | | *30.642* | | | |
| **OBS-SV24** | **Negative response** | **Count** | 31_a_ | 38_a, b_ | 58_b_ | 127 |
|  |  | **% within diagnosis** | 72.1% | 90.5% | 96.7% | 87.6% |
|  | **Positive**  **response** | **Count** | 12_a_ | 4_a, b_ | 2_b_ | 18 |
|  |  | **% within diagnosis** | 27.9% | 9.5% | 3.3% | 12.4% |
| ***Pearson Chi-Square*** | | | *14.366* | | | |
| ***Likelihood Ratio*** | | | *13.903* | | | |
| ***Linear-by-Linear Association*** | | | *13.262* | | | |
| **OBS-SV25** | **Negative response** | **Count** | 16_a_ | 28_b_ | 52_c_ | 96 |
|  |  | **% within diagnosis** | 37.2% | 66.7% | 86.7% | 66.2% |
|  | **Positive**  **response** | **Count** | 27_a_ | 14_b_ | 8_c_ | 49 |
|  |  | **% within diagnosis** | 62.8% | 33.3% | 13.3% | 33.8% |
| ***Pearson Chi-Square*** | | | *27.391* | | | |
| ***Likelihood Ratio*** | | | *28.146* | | | |
| ***Linear-by-Linear Association*** | | | *26.908* | | | |
| **OBS-SV26** | **Negative response** | **Count** | 22_a_ | 30_a_ | 60_b_ | 112 |
|  |  | **% within diagnosis** | 51.2% | 71.4% | 100.0% | 77.2% |
|  | **Positive**  **response** | **Count** | 21_a_ | 12_a_ | 0_b_ | 33 |
|  |  | **% within diagnosis** | 48.8% | 28.6% | 0.0% | 22.8% |
| ***Pearson Chi-Square*** | | | *35.122* | | | |
| ***Likelihood Ratio*** | | | *45.697* | | | |
| ***Linear-by-Linear Association*** | | | *34.591* | | | |
| **OBS-SV27** | **Negative response** | **Count** | 17_a_ | 32_b_ | 60_c_ | 109 |
|  |  | **% within diagnosis** | 39.5% | 76.2% | 100.0% | 75.2% |
|  | **Positive**  **response** | **Count** | 26_a_ | 10_b_ | 0_c_ | 36 |
|  |  | **% within diagnosis** | 60.5% | 23.8% | 0.0% | 24.8% |
| ***Pearson Chi-Square*** | | | *49.101* | | | |
| ***Likelihood Ratio*** | | | *58.707* | | | |
| ***Linear-by-Linear Association*** | | | *48.113* | | | |
| **OBS-SV28** | **Negative response** | **Count** | 21_a_ | 30_a, b_ | 53_b_ | 104 |
|  |  | **% within diagnosis** | 48.8% | 71.4% | 88.3% | 71.7% |
|  | **Positive**  **response** | **Count** | 22_a_ | 12_a, b_ | 7_b_ | 41 |
|  |  | **% within diagnosis** | 51.2% | 28.6% | 11.7% | 28.3% |
| ***Pearson Chi-Square*** | | | *19.269* | | | |
| ***Likelihood Ratio*** | | | *19.637* | | | |
| ***Linear-by-Linear Association*** | | | *19.019* | | | |
| **OBS-SV29** | **Negative response** | **Count** | 25_a_ | 32_a, b_ | 55_b_ | 112 |
|  |  | **% within diagnosis** | 58.1% | 76.2% | 91.7% | 77.2% |
|  | **Positive**  **response** | **Count** | 18_a_ | 10_a, b_ | 5_b_ | 33 |
|  |  | **% within diagnosis** | 41.9% | 23.8% | 8.3% | 22.8% |
| ***Pearson Chi-Square*** | | | *16.054* | | | |
| ***Likelihood Ratio*** | | | *16.548* | | | |
| ***Linear-by-Linear Association*** | | | *15.916* | | | |
| **OBS-SV30** | **Negative response** | **Count** | 27_a_ | 31_a_ | 57_b_ | 115 |
|  |  | **% within diagnosis** | 62.8% | 73.8% | 95.0% | 79.3% |
|  | **Positive**  **response** | **Count** | 16_a_ | 11_a_ | 3_b_ | 30 |
|  |  | **% within diagnosis** | 37.2% | 26.2% | 5.0% | 20.7% |
| ***Pearson Chi-Square*** | | | *16.927* | | | |
| ***Likelihood Ratio*** | | | *18.956* | | | |
| ***Linear-by-Linear Association*** | | | *16.347* | | | |
| **OBS-SV31** | **Negative response** | **Count** | 12_a_ | 33_b_ | 53_b_ | 98 |
|  |  | **% within diagnosis** | 27.9% | 78.6% | 88.3% | 67.6% |
|  | **Positive**  **response** | **Count** | 31_a_ | 9_b_ | 7_b_ | 47 |
|  |  | **% within diagnosis** | 72.1% | 21.4% | 11.7% | 32.4% |
| ***Pearson Chi-Square*** | | | *45.006* | | | |
| ***Likelihood Ratio*** | | | *44.895* | | | |
| ***Linear-by-Linear Association*** | | | *39.084* | | | |
| **OBS-SV32** | **Negative response** | **Count** | 25_a_ | 35_b_ | 55_b_ | 115 |
|  |  | **% within diagnosis** | 58.1% | 83.3% | 91.7% | 79.3% |
|  | **Positive**  **response** | **Count** | 18_a_ | 7_b_ | 5_b_ | 30 |
|  |  | **% within diagnosis** | 41.9% | 16.7% | 8.3% | 20.7% |
| ***Pearson Chi-Square*** | | | *17.742* | | | |
| ***Likelihood Ratio*** | | | *17.113* | | | |
| ***Linear-by-Linear Association*** | | | *16.347* | | | |
| **OBS-SV33** | **Negative response** | **Count** | 27_a_ | 37_b_ | 56_b_ | 120 |
|  |  | **% within diagnosis** | 62.8% | 88.1% | 93.3% | 82.8% |
|  | **Positive**  **response** | **Count** | 16_a_ | 5_b_ | 4_b_ | 25 |
|  |  | **% within diagnosis** | 37.2% | 11.9% | 6.7% | 17.2% |
| ***Pearson Chi-Square*** | | | *17.556* | | | |
| ***Likelihood Ratio*** | | | *16.492* | | | |
| ***Linear-by-Linear Association*** | | | *15.362* | | | |
| **OBS-SV34** | **Negative response** | **Count** | 20_a_ | 34_b_ | 55_b_ | 109 |
|  |  | **% within diagnosis** | 46.5% | 81.0% | 91.7% | 75.2% |
|  | **Positive**  **response** | **Count** | 23_a_ | 8_b_ | 5_b_ | 36 |
|  |  | **% within diagnosis** | 53.5% | 19.0% | 8.3% | 24.8% |
| ***Pearson Chi-Square*** | | | *28.424* | | | |
| ***Likelihood Ratio*** | | | *27.803* | | | |
| ***Linear-by-Linear Association*** | | | *26.012* | | | |
| **OBS-SV35** | **Negative response** | **Count** | 21_a_ | 37_b_ | 57_b_ | 115 |
|  |  | **% within diagnosis** | 48.8% | 88.1% | 95.0% | 79.3% |
|  | **Positive**  **response** | **Count** | 22_a_ | 5_b_ | 3_b_ | 30 |
|  |  | **% within diagnosis** | 51.2% | 11.9% | 5.0% | 20.7% |
| ***Pearson Chi-Square*** | | | *35.311* | | | |
| ***Likelihood Ratio*** | | | *33.775* | | | |
| ***Linear-by-Linear Association*** | | | *30.380* | | | |
| **OBS-SV36** | **Negative response** | **Count** | 20_a_ | 37_b_ | 56_b_ | 113 |
|  |  | **% within diagnosis** | 46.5% | 88.1% | 93.3% | 77.9% |
|  | **Positive**  **response** | **Count** | 23_a_ | 5_b_ | 4_b_ | 32 |
|  |  | **% within diagnosis** | 53.5% | 11.9% | 6.7% | 22.1% |
| ***Pearson Chi-Square*** | | | *35.481* | | | |
| ***Likelihood Ratio*** | | | *33.601* | | | |
| ***Linear-by-Linear Association*** | | | *29.592* | | | |
| **OBS-SV37** | **Negative response** | **Count** | 17_a_ | 28_b_ | 54_c_ | 99 |
|  |  | **% within diagnosis** | 39.5% | 66.7% | 90.0% | 68.3% |
|  | **Positive**  **response** | **Count** | 26_a_ | 14_b_ | 6_c_ | 46 |
|  |  | **% within diagnosis** | 60.5% | 33.3% | 10.0% | 31.7% |
| ***Pearson Chi-Square*** | | | *29.522* | | | |
| ***Likelihood Ratio*** | | | *30.994* | | | |
| ***Linear-by-Linear Association*** | | | *29.270* | | | |
| **OBS-SV38** | **Negative response** | **Count** | 27_a_ | 34_a, b_ | 57_b_ | 118 |
|  |  | **% within diagnosis** | 62.8% | 81.0% | 95.0% | 81.4% |
|  | **Positive**  **response** | **Count** | 16_a_ | 8_a, b_ | 3_b_ | 27 |
|  |  | **% within diagnosis** | 37.2% | 19.0% | 5.0% | 18.6% |
| ***Pearson Chi-Square*** | | | *17.156* | | | |
| ***Likelihood Ratio*** | | | *17.908* | | | |
| ***Linear-by-Linear Association*** | | | *16.956* | | | |
| **OBS-SV39** | **Negative response** | **Count** | 26_a_ | 29_a_ | 53_b_ | 108 |
|  |  | **% within diagnosis** | 60.5% | 69.0% | 88.3% | 74.5% |
|  | **Positive**  **response** | **Count** | 17_a_ | 13_a_ | 7_b_ | 37 |
|  |  | **% within diagnosis** | 39.5% | 31.0% | 11.7% | 25.5% |
| ***Pearson Chi-Square*** | | | *11.155* | | | |
| ***Likelihood Ratio*** | | | *11.792* | | | |
| ***Linear-by-Linear Association*** | | | *10.635* | | | |
| **OBS-SV40** | **Negative response** | **Count** | 34_a_ | 31_a_ | 52_a_ | 117 |
|  |  | **% within diagnosis** | 79.1% | 73.8% | 86.7% | 80.7% |
|  | **Positive**  **response** | **Count** | 9_a_ | 11_a_ | 8_a_ | 28 |
|  |  | **% within diagnosis** | 20.9% | 26.2% | 13.3% | 19.3% |
| ***Pearson Chi-Square*** | | | *2.724* | | | |
| ***Likelihood Ratio*** | | | *2.756* | | | |
| ***Linear-by-Linear Association*** | | | *1.157* | | | |
| **OBS-SV41** | **Negative response** | **Count** | 30_a_ | 36_a_ | 52_a_ | 118 |
|  |  | **% within diagnosis** | 69.8% | 85.7% | 86.7% | 81.4% |
|  | **Positive**  **response** | **Count** | 13_a_ | 6_a_ | 8_a_ | 27 |
|  |  | **% within diagnosis** | 30.2% | 14.3% | 13.3% | 18.6% |
| ***Pearson Chi-Square*** | | | *5.454* | | | |
| ***Likelihood Ratio*** | | | *5.123* | | | |
| ***Linear-by-Linear Association*** | | | *4.326* | | | |
| **OBS-SV42** | **Negative response** | **Count** | 25_a_ | 29_a, b_ | 52_b_ | 106 |
|  |  | **% within diagnosis** | 58.1% | 69.0% | 86.7% | 73.1% |
|  | **Positive**  **response** | **Count** | 18_a_ | 13_a, b_ | 8_b_ | 39 |
|  |  | **% within diagnosis** | 41.9% | 31.0% | 13.3% | 26.9% |
| ***Pearson Chi-Square*** | | | *10.862* | | | |
| ***Likelihood Ratio*** | | | *11.287* | | | |
| ***Linear-by-Linear Association*** | | | *10.619* | | | |
| **OBS-SV43** | **Negative response** | **Count** | 28_a_ | 39_b_ | 55_b_ | 122 |
|  |  | **% within diagnosis** | 65.1% | 92.9% | 91.7% | 84.1% |
|  | **Positive**  **response** | **Count** | 15_a_ | 3_b_ | 5_b_ | 23 |
|  |  | **% within diagnosis** | 34.9% | 7.1% | 8.3% | 15.9% |
| ***Pearson Chi-Square*** | | | *16.598* | | | |
| ***Likelihood Ratio*** | | | *15.186* | | | |
| ***Linear-by-Linear Association*** | | | *11.876* | | | |
| **OBS-SV44** | **Negative response** | **Count** | 27_a_ | 39_b_ | 57_b_ | 123 |
|  |  | **% within diagnosis** | 62.8% | 92.9% | 95.0% | 84.8% |
|  | **Positive**  **response** | **Count** | 16_a_ | 3_b_ | 3_b_ | 22 |
|  |  | **% within diagnosis** | 37.2% | 7.1% | 5.0% | 15.2% |
| ***Pearson Chi-Square*** | | | *23.153* | | | |
| ***Likelihood Ratio*** | | | *21.248* | | | |
| ***Linear-by-Linear Association*** | | | *18.542* | | | |
| **OBS-SV45** | **Negative response** | **Count** | 25_a_ | 39_b_ | 58_b_ | 122 |
|  |  | **% within diagnosis** | 58.1% | 92.9% | 96.7% | 84.1% |
|  | **Positive**  **response** | **Count** | 18_a_ | 3_b_ | 2_b_ | 23 |
|  |  | **% within diagnosis** | 41.9% | 7.1% | 3.3% | 15.9% |
| ***Pearson Chi-Square*** | | | *31.227* | | | |
| ***Likelihood Ratio*** | | | *29.221* | | | |
| ***Linear-by-Linear Association*** | | | *25.752* | | | |
| **OBS-SV46** | **Negative response** | **Count** | 26_a_ | 37_b_ | 55_b_ | 118 |
|  |  | **% within diagnosis** | 60.5% | 88.1% | 91.7% | 81.4% |
|  | **Positive**  **response** | **Count** | 17_a_ | 5_b_ | 5_b_ | 27 |
|  |  | **% within diagnosis** | 39.5% | 11.9% | 8.3% | 18.6% |
| ***Pearson Chi-Square*** | | | *17.852* | | | |
| ***Likelihood Ratio*** | | | *16.601* | | | |
| ***Linear-by-Linear Association*** | | | *14.923* | | | |
| **OBS-SV47** | **Negative response** | **Count** | 28_a_ | 38_b_ | 54_b_ | 120 |
|  |  | **% within diagnosis** | 65.1% | 90.5% | 90.0% | 82.8% |
|  | **Positive**  **response** | **Count** | 15_a_ | 4_b_ | 6_b_ | 25 |
|  |  | **% within diagnosis** | 34.9% | 9.5% | 10.0% | 17.2% |
| ***Pearson Chi-Square*** | | | *13.338* | | | |
| ***Likelihood Ratio*** | | | *12.265* | | | |
| ***Linear-by-Linear Association*** | | | *9.809* | | | |
| **OBS-SV48** | **Negative response** | **Count** | 20_a_ | 27_a_ | 51_b_ | 98 |
|  |  | **% within diagnosis** | 46.5% | 64.3% | 85.0% | 67.6% |
|  | **Positive**  **response** | **Count** | 23_a_ | 15_a_ | 9_b_ | 47 |
|  |  | **% within diagnosis** | 53.5% | 35.7% | 15.0% | 32.4% |
| ***Pearson Chi-Square*** | | | *17.232* | | | |
| ***Likelihood Ratio*** | | | *17.811* | | | |
| ***Linear-by-Linear Association*** | | | *17.084* | | | |
| **OBS-SV49** | **Negative response** | **Count** | 24_a_ | 31_a, b_ | 54_b_ | 109 |
|  |  | **% within diagnosis** | 55.8% | 73.8% | 90.0% | 75.2% |
|  | **Positive**  **response** | **Count** | 19_a_ | 11_a, b_ | 6_b_ | 36 |
|  |  | **% within diagnosis** | 44.2% | 26.2% | 10.0% | 24.8% |
| ***Pearson Chi-Square*** | | | *15.744* | | | |
| ***Likelihood Ratio*** | | | *16.184* | | | |
| ***Linear-by-Linear Association*** | | | *15.623* | | | |
| **OBS-SV50** | **Negative response** | **Count** | 13_a_ | 28_b_ | 58_c_ | 99 |
|  |  | **% within diagnosis** | 30.2% | 66.7% | 96.7% | 68.3% |
|  | **Positive**  **response** | **Count** | 30_a_ | 14_b_ | 2_c_ | 46 |
|  |  | **% within diagnosis** | 69.8% | 33.3% | 3.3% | 31.7% |
| ***Pearson Chi-Square*** | | | *51.110* | | | |
| ***Likelihood Ratio*** | | | *57.477* | | | |
| ***Linear-by-Linear Association*** | | | *50.617* | | | |
| **OBS-SV51** | **Negative response** | **Count** | 19_a_ | 29_a_ | 56_b_ | 104 |
|  |  | **% within diagnosis** | 44.2% | 69.0% | 93.3% | 71.7% |
|  | **Positive**  **response** | **Count** | 24_a_ | 13_a_ | 4_b_ | 41 |
|  |  | **% within diagnosis** | 55.8% | 31.0% | 6.7% | 28.3% |
| ***Pearson Chi-Square*** | | | *30.042* | | | |
| ***Likelihood Ratio*** | | | *32.315* | | | |
| ***Linear-by-Linear Association*** | | | *29.834* | | | |
| **OBS-SV52** | **Negative response** | **Count** | 22_a_ | 34_b_ | 57_b_ | 113 |
|  |  | **% within diagnosis** | 51.2% | 81.0% | 95.0% | 77.9% |
|  | **Positive**  **response** | **Count** | 21_a_ | 8_b_ | 3_b_ | 32 |
|  |  | **% within diagnosis** | 48.8% | 19.0% | 5.0% | 22.1% |
| ***Pearson Chi-Square*** | | | *28.302* | | | |
| ***Likelihood Ratio*** | | | *28.746* | | | |
| ***Linear-by-Linear Association*** | | | *27.048* | | | |
| **OBS-SV53** | **Negative response** | **Count** | 24_a_ | 31_a, b_ | 53_b_ | 108 |
|  |  | **% within diagnosis** | 55.8% | 73.8% | 88.3% | 74.5% |
|  | **Positive**  **response** | **Count** | 19_a_ | 11_a, b_ | 7_b_ | 37 |
|  |  | **% within diagnosis** | 44.2% | 26.2% | 11.7% | 25.5% |
| ***Pearson Chi-Square*** | | | *13.951* | | | |
| ***Likelihood Ratio*** | | | *14.146* | | | |
| ***Linear-by-Linear Association*** | | | *13.809* | | | |
| **OBS-SV54** | **Negative response** | **Count** | 21_a_ | 38_b_ | 52_b_ | 111 |
|  |  | **% within diagnosis** | 48.8% | 90.5% | 86.7% | 76.6% |
|  | **Positive**  **response** | **Count** | 22_a_ | 4_b_ | 8_b_ | 34 |
|  |  | **% within diagnosis** | 51.2% | 9.5% | 13.3% | 23.4% |
| ***Pearson Chi-Square*** | | | *26.357* | | | |
| ***Likelihood Ratio*** | | | *24.819* | | | |
| ***Linear-by-Linear Association*** | | | *17.720* | | | |
| **OBS-SV55** | **Negative response** | **Count** | 14_a_ | 28_b_ | 53_c_ | 95 |
|  |  | **% within diagnosis** | 32.6% | 66.7% | 88.3% | 65.5% |
|  | **Positive**  **response** | **Count** | 29_a_ | 14_b_ | 7_c_ | 50 |
|  |  | **% within diagnosis** | 67.4% | 33.3% | 11.7% | 34.5% |
| ***Pearson Chi-Square*** | | | *34.526* | | | |
| ***Likelihood Ratio*** | | | *35.853* | | | |
| ***Linear-by-Linear Association*** | | | *33.784* | | | |
| **OBS-SV56** | **Negative response** | **Count** | 14_a_ | 33_b_ | 52_b_ | 99 |
|  |  | **% within diagnosis** | 32.6% | 78.6% | 86.7% | 68.3% |
|  | **Positive**  **response** | **Count** | 29_a_ | 9_b_ | 8_b_ | 46 |
|  |  | **% within diagnosis** | 67.4% | 21.4% | 13.3% | 31.7% |
| ***Pearson Chi-Square*** | | | *36.751* | | | |
| ***Likelihood Ratio*** | | | *36.152* | | | |
| ***Linear-by-Linear Association*** | | | *31.620* | | | |
| **OBS-SV57** | **Negative response** | **Count** | 17_a_ | 26_a_ | 54_b_ | 97 |
|  |  | **% within diagnosis** | 39.5% | 61.9% | 90.0% | 66.9% |
|  | **Positive**  **response** | **Count** | 26_a_ | 16_a_ | 6_b_ | 48 |
|  |  | **% within diagnosis** | 60.5% | 38.1% | 10.0% | 33.1% |
| ***Pearson Chi-Square*** | | | *29.472* | | | |
| ***Likelihood Ratio*** | | | *31.580* | | | |
| ***Linear-by-Linear Association*** | | | *29.160* | | | |
| **OBS-SV58** | **Negative response** | **Count** | 24_a_ | 35_b_ | 51_b_ | 110 |
|  |  | **% within diagnosis** | 55.8% | 83.3% | 85.0% | 75.9% |
|  | **Positive**  **response** | **Count** | 19_a_ | 7_b_ | 9_b_ | 35 |
|  |  | **% within diagnosis** | 44.2% | 16.7% | 15.0% | 24.1% |
| ***Pearson Chi-Square*** | | | *13.455* | | | |
| ***Likelihood Ratio*** | | | *12.673* | | | |
| ***Linear-by-Linear Association*** | | | *10.680* | | | |
| **OBS-SV59** | **Negative response** | **Count** | 21_a_ | 33_b_ | 58_c_ | 112 |
|  |  | **% within diagnosis** | 48.8% | 78.6% | 96.7% | 77.2% |
|  | **Positive**  **response** | **Count** | 22_a_ | 9_b_ | 2_c_ | 33 |
|  |  | **% within diagnosis** | 51.2% | 21.4% | 3.3% | 22.8% |
| ***Pearson Chi-Square*** | | | *32.656* | | | |
| ***Likelihood Ratio*** | | | *34.770* | | | |
| ***Linear-by-Linear Association*** | | | *31.865* | | | |
| **OBS-SV60** | **Negative response** | **Count** | 22_a_ | 40_b_ | 58_b_ | 120 |
|  |  | **% within diagnosis** | 51.2% | 95.2% | 96.7% | 82.8% |
|  | **Positive**  **response** | **Count** | 21_a_ | 2_b_ | 2_b_ | 25 |
|  |  | **% within diagnosis** | 48.8% | 4.8% | 3.3% | 17.2% |
| ***Pearson Chi-Square*** | | | *42.803* | | | |
| ***Likelihood Ratio*** | | | *40.105* | | | |
| ***Linear-by-Linear Association*** | | | *33.142* | | | |
| **OBS-SV61** | **Negative response** | **Count** | 25_a_ | 34_a, b_ | 55_b_ | 114 |
|  |  | **% within diagnosis** | 58.1% | 81.0% | 91.7% | 78.6% |
|  | **Positive**  **response** | **Count** | 18_a_ | 8_a, b_ | 5_b_ | 31 |
|  |  | **% within diagnosis** | 41.9% | 19.0% | 8.3% | 21.4% |
| ***Pearson Chi-Square*** | | | *16.942* | | | |
| ***Likelihood Ratio*** | | | *16.705* | | | |
| ***Linear-by-Linear Association*** | | | *16.186* | | | |
| **OBS-SV62** | **Negative response** | **Count** | 28_a_ | 39_b_ | 57_b_ | 124 |
|  |  | **% within diagnosis** | 65.1% | 92.9% | 95.0% | 85.5% |
|  | **Positive**  **response** | **Count** | 15_a_ | 3_b_ | 3_b_ | 21 |
|  |  | **% within diagnosis** | 34.9% | 7.1% | 5.0% | 14.5% |
| ***Pearson Chi-Square*** | | | *20.633* | | | |
| ***Likelihood Ratio*** | | | *18.898* | | | |
| ***Linear-by-Linear Association*** | | | *16.603* | | | |
| **OBS-SV63** | **Negative response** | **Count** | 22_a_ | 33_b_ | 55_b_ | 110 |
|  |  | **% within diagnosis** | 51.2% | 78.6% | 91.7% | 75.9% |
|  | **Positive**  **response** | **Count** | 21_a_ | 9_b_ | 5_b_ | 35 |
|  |  | **% within diagnosis** | 48.8% | 21.4% | 8.3% | 24.1% |
| ***Pearson Chi-Square*** | | | *22.678* | | | |
| ***Likelihood Ratio*** | | | *22.620* | | | |
| ***Linear-by-Linear Association*** | | | *21.700* | | | |
| **OBS-SV64** | **Negative response** | **Count** | 27_a_ | 39_b_ | 58_b_ | 124 |
|  |  | **% within diagnosis** | 62.8% | 92.9% | 96.7% | 85.5% |
|  | **Positive**  **response** | **Count** | 16_a_ | 3_b_ | 2_b_ | 21 |
|  |  | **% within diagnosis** | 37.2% | 7.1% | 3.3% | 14.5% |
| ***Pearson Chi-Square*** | | | *25.781* | | | |
| ***Likelihood Ratio*** | | | *24.036* | | | |
| ***Linear-by-Linear Association*** | | | *21.513* | | | |
| **OBS-SV65** | **Negative response** | **Count** | 26_a_ | 39_b_ | 56_b_ | 121 |
|  |  | **% within diagnosis** | 60.5% | 92.9% | 93.3% | 83.4% |
|  | **Positive**  **response** | **Count** | 17_a_ | 3_b_ | 4_b_ | 24 |
|  |  | **% within diagnosis** | 39.5% | 7.1% | 6.7% | 16.6% |
| ***Pearson Chi-Square*** | | | *23.381* | | | |
| ***Likelihood Ratio*** | | | *21.406* | | | |
| ***Linear-by-Linear Association*** | | | *17.801* | | | |
| **OBS-SV66** | **Negative response** | **Count** | 19_a_ | 28_a_ | 57_b_ | 104 |
|  |  | **% within diagnosis** | 44.2% | 66.7% | 95.0% | 71.7% |
|  | **Positive**  **response** | **Count** | 24_a_ | 14_a_ | 3_b_ | 41 |
|  |  | **% within diagnosis** | 55.8% | 33.3% | 5.0% | 28.3% |
| ***Pearson Chi-Square*** | | | *32.637* | | | |
| ***Likelihood Ratio*** | | | *36.390* | | | |
| ***Linear-by-Linear Association*** | | | *32.287* | | | |
| **OBS-SV67** | **Negative response** | **Count** | 24_a_ | 38_b_ | 56_b_ | 118 |
|  |  | **% within diagnosis** | 55.8% | 90.5% | 93.3% | 81.4% |
|  | **Positive**  **response** | **Count** | 19_a_ | 4_b_ | 4_b_ | 27 |
|  |  | **% within diagnosis** | 44.2% | 9.5% | 6.7% | 18.6% |
| ***Pearson Chi-Square*** | | | *26.498* | | | |
| ***Likelihood Ratio*** | | | *24.559* | | | |
| ***Linear-by-Linear Association*** | | | *21.411* | | | |
| **OBS-SV68** | **Negative response** | **Count** | 29_a_ | 36_a, b_ | 54_b_ | 119 |
|  |  | **% within diagnosis** | 67.4% | 85.7% | 90.0% | 82.1% |
|  | **Positive**  **response** | **Count** | 14_a_ | 6_a, b_ | 6_b_ | 26 |
|  |  | **% within diagnosis** | 32.6% | 14.3% | 10.0% | 17.9% |
| ***Pearson Chi-Square*** | | | *9.196* | | | |
| ***Likelihood Ratio*** | | | *8.674* | | | |
| ***Linear-by-Linear Association*** | | | *8.155* | | | |
| **OBS-SV69** | **Negative response** | **Count** | 34_a_ | 35_a, b_ | 57_b_ | 126 |
|  |  | **% within diagnosis** | 79.1% | 83.3% | 95.0% | 86.9% |
|  | **Positive**  **response** | **Count** | 9_a_ | 7_a, b_ | 3_b_ | 19 |
|  |  | **% within diagnosis** | 20.9% | 16.7% | 5.0% | 13.1% |
| ***Pearson Chi-Square*** | | | *6.242* | | | |
| ***Likelihood Ratio*** | | | *6.831* | | | |
| ***Linear-by-Linear Association*** | | | *5.845* | | | |
| **OBS-SV70** | **Negative response** | **Count** | 22_a_ | 34_b_ | 56_b_ | 112 |
|  |  | **% within diagnosis** | 51.2% | 81.0% | 93.3% | 77.2% |
|  | **Positive**  **response** | **Count** | 21_a_ | 8_b_ | 4_b_ | 33 |
|  |  | **% within diagnosis** | 48.8% | 19.0% | 6.7% | 22.8% |
| ***Pearson Chi-Square*** | | | *25.803* | | | |
| ***Likelihood Ratio*** | | | *25.660* | | | |
| ***Linear-by-Linear Association*** | | | *24.358* | | | |
| **OBS-SV71** | **Negative response** | **Count** | 14_a_ | 33_b_ | 57_c_ | 104 |
|  |  | **% within diagnosis** | 32.6% | 78.6% | 95.0% | 71.7% |
|  | **Positive**  **response** | **Count** | 29_a_ | 9_b_ | 3_c_ | 41 |
|  |  | **% within diagnosis** | 67.4% | 21.4% | 5.0% | 28.3% |
| ***Pearson Chi-Square*** | | | *49.523* | | | |
| ***Likelihood Ratio*** | | | *50.974* | | | |
| ***Linear-by-Linear Association*** | | | *46.011* | | | |
| **OBS-SV72** | **Negative response** | **Count** | 17_a_ | 36_b_ | 53_b_ | 106 |
|  |  | **% within diagnosis** | 39.5% | 85.7% | 88.3% | 73.1% |
|  | **Positive**  **response** | **Count** | 26_a_ | 6_b_ | 7_b_ | 39 |
|  |  | **% within diagnosis** | 60.5% | 14.3% | 11.7% | 26.9% |
| ***Pearson Chi-Square*** | | | *35.118* | | | |
| ***Likelihood Ratio*** | | | *33.456* | | | |
| ***Linear-by-Linear Association*** | | | *27.785* | | | |
| **OBS-SV73** | **Negative response** | **Count** | 26_a_ | 42_b_ | 56_b_ | 124 |
|  |  | **% within diagnosis** | 60.5% | 100.0% | 93.3% | 85.5% |
|  | **Positive**  **response** | **Count** | 17_a_ | 0_b_ | 4_b_ | 21 |
|  |  | **% within diagnosis** | 39.5% | 0.0% | 6.7% | 14.5% |
| ***Pearson Chi-Square*** | | | *31.862* | | | |
| ***Likelihood Ratio*** | | | *32.848* | | | |
| ***Linear-by-Linear Association*** | | | *18.979* | | | |
| **OBS-SV74** | **Negative response** | **Count** | 28_a_ | 36_a, b_ | 55_b_ | 119 |
|  |  | **% within diagnosis** | 65.1% | 85.7% | 91.7% | 82.1% |
|  | **Positive**  **response** | **Count** | 15_a_ | 6_a, b_ | 5_b_ | 26 |
|  |  | **% within diagnosis** | 34.9% | 14.3% | 8.3% | 17.9% |
| ***Pearson Chi-Square*** | | | *12.533* | | | |
| ***Likelihood Ratio*** | | | *11.912* | | | |
| ***Linear-by-Linear Association*** | | | *11.375* | | | |
| **OBS-SV75** | **Negative response** | **Count** | 25_a_ | 38_b_ | 56_b_ | 119 |
|  |  | **% within diagnosis** | 58.1% | 90.5% | 93.3% | 82.1% |
|  | **Positive**  **response** | **Count** | 18_a_ | 4_b_ | 4_b_ | 26 |
|  |  | **% within diagnosis** | 41.9% | 9.5% | 6.7% | 17.9% |
| ***Pearson Chi-Square*** | | | *23.923* | | | |
| ***Likelihood Ratio*** | | | *22.125* | | | |
| ***Linear-by-Linear Association*** | | | *19.419* | | | |
| **OBS-SV76** | **Negative response** | **Count** | 21_a_ | 39_b_ | 57_b_ | 117 |
|  |  | **% within diagnosis** | 48.8% | 92.9% | 95.0% | 80.7% |
|  | **Positive**  **response** | **Count** | 22_a_ | 3_b_ | 3_b_ | 28 |
|  |  | **% within diagnosis** | 51.2% | 7.1% | 5.0% | 19.3% |
| ***Pearson Chi-Square*** | | | *39.876* | | | |
| ***Likelihood Ratio*** | | | *37.277* | | | |
| ***Linear-by-Linear Association*** | | | *31.331* | | | |
| **OBS-SV77** | **Negative response** | **Count** | 27_a_ | 40_b_ | 57_b_ | 124 |
|  |  | **% within diagnosis** | 62.8% | 95.2% | 95.0% | 85.5% |
|  | **Positive**  **response** | **Count** | 16_a_ | 2_b_ | 3_b_ | 21 |
|  |  | **% within diagnosis** | 37.2% | 4.8% | 5.0% | 14.5% |
| ***Pearson Chi-Square*** | | | *25.493* | | | |
| ***Likelihood Ratio*** | | | *23.285* | | | |
| ***Linear-by-Linear Association*** | | | *18.979* | | | |
| **OBS-SV78** | **Negative response** | **Count** | 25_a_ | 36_b_ | 60_c_ | 121 |
|  |  | **% within diagnosis** | 58.1% | 85.7% | 100.0% | 83.4% |
|  | **Positive**  **response** | **Count** | 18_a_ | 6_b_ | 0_c_ | 24 |
|  |  | **% within diagnosis** | 41.9% | 14.3% | 0.0% | 16.6% |
| ***Pearson Chi-Square*** | | | *31.998* | | | |
| ***Likelihood Ratio*** | | | *37.209* | | | |
| ***Linear-by-Linear Association*** | | | *30.838* | | | |
| **OBS-SV79** | **Negative response** | **Count** | 21_a_ | 40_b_ | 57_b_ | 118 |
|  |  | **% within diagnosis** | 48.8% | 95.2% | 95.0% | 81.4% |
|  | **Positive**  **response** | **Count** | 22_a_ | 2_b_ | 3_b_ | 27 |
|  |  | **% within diagnosis** | 51.2% | 4.8% | 5.0% | 18.6% |
| ***Pearson Chi-Square*** | | | *42.720* | | | |
| ***Likelihood Ratio*** | | | *39.905* | | | |
| ***Linear-by-Linear Association*** | | | *31.878* | | | |
| **OBS-SV80** | **Negative response** | **Count** | 30_a_ | 37_a, b_ | 58_b_ | 125 |
|  |  | **% within diagnosis** | 69.8% | 88.1% | 96.7% | 86.2% |
|  | **Positive**  **response** | **Count** | 13_a_ | 5_a, b_ | 2_b_ | 20 |
|  |  | **% within diagnosis** | 30.2% | 11.9% | 3.3% | 13.8% |
| ***Pearson Chi-Square*** | | | *15.420* | | | |
| ***Likelihood Ratio*** | | | *15.443* | | | |
| ***Linear-by-Linear Association*** | | | *14.725* | | | |
| **OBS-SV81** | **Negative response** | **Count** | 28_a_ | 37_b_ | 53_b_ | 118 |
|  |  | **% within diagnosis** | 65.1% | 88.1% | 88.3% | 81.4% |
|  | **Positive**  **response** | **Count** | 15_a_ | 5_b_ | 7_b_ | 27 |
|  |  | **% within diagnosis** | 34.9% | 11.9% | 11.7% | 18.6% |
| ***Pearson Chi-Square*** | | | *10.670* | | | |
| ***Likelihood Ratio*** | | | *9.888* | | | |
| ***Linear-by-Linear Association*** | | | *8.089* | | | |
| **OBS-SV82** | **Negative response** | **Count** | 26_a_ | 35_a, b_ | 55_b_ | 116 |
|  |  | **% within diagnosis** | 60.5% | 83.3% | 91.7% | 80.0% |
|  | **Positive**  **response** | **Count** | 17_a_ | 7_a, b_ | 5_b_ | 29 |
|  |  | **% within diagnosis** | 39.5% | 16.7% | 8.3% | 20.0% |
| ***Pearson Chi-Square*** | | | *15.652* | | | |
| ***Likelihood Ratio*** | | | *15.136* | | | |
| ***Linear-by-Linear Association*** | | | *14.574* | | | |
| **OBS-SV83** | **Negative response** | **Count** | 26_a_ | 39_b_ | 56_b_ | 121 |
|  |  | **% within diagnosis** | 60.5% | 92.9% | 93.3% | 83.4% |
|  | **Positive**  **response** | **Count** | 17_a_ | 3_b_ | 4_b_ | 24 |
|  |  | **% within diagnosis** | 39.5% | 7.1% | 6.7% | 16.6% |
| ***Pearson Chi-Square*** | | | *23.381* | | | |
| ***Likelihood Ratio*** | | | *21.406* | | | |
| ***Linear-by-Linear Association*** | | | *17.801* | | | |
| **OBS-SV84** | **Negative response** | **Count** | 23_a_ | 36_b_ | 59_c_ | 118 |
|  |  | **% within diagnosis** | 53.5% | 85.7% | 98.3% | 81.4% |
|  | **Positive**  **response** | **Count** | 20_a_ | 6_b_ | 1_c_ | 27 |
|  |  | **% within diagnosis** | 46.5% | 14.3% | 1.7% | 18.6% |
| ***Pearson Chi-Square*** | | | *33.976* | | | |
| ***Likelihood Ratio*** | | | *35.373* | | | |
| ***Linear-by-Linear Association*** | | | *31.878* | | | |
| **OBS-SV85** | **Negative response** | **Count** | 23_a_ | 38_b_ | 55_b_ | 116 |
|  |  | **% within diagnosis** | 53.5% | 90.5% | 91.7% | 80.0% |
|  | **Positive**  **response** | **Count** | 20_a_ | 4_b_ | 5_b_ | 29 |
|  |  | **% within diagnosis** | 46.5% | 9.5% | 8.3% | 20.0% |
| ***Pearson Chi-Square*** | | | *26.875* | | | |
| ***Likelihood Ratio*** | | | *24.878* | | | |
| ***Linear-by-Linear Association*** | | | *20.805* | | | |
| **OBS-SV86** | **Negative response** | **Count** | 19_a_ | 39_b_ | 60_b_ | 118 |
|  |  | **% within diagnosis** | 44.2% | 92.9% | 100.0% | 81.4% |
|  | **Positive**  **response** | **Count** | 24_a_ | 3_b_ | 0_b_ | 27 |
|  |  | **% within diagnosis** | 55.8% | 7.1% | 0.0% | 18.6% |
| ***Pearson Chi-Square*** | | | *56.635* | | | |
| ***Likelihood Ratio*** | | | *58.753* | | | |
| ***Linear-by-Linear Association*** | | | *47.882* | | | |
| **OBS-SV87** | **Negative response** | **Count** | 21_a_ | 38_b_ | 57_b_ | 116 |
|  |  | **% within diagnosis** | 48.8% | 90.5% | 95.0% | 80.0% |
|  | **Positive**  **response** | **Count** | 22_a_ | 4_b_ | 3_b_ | 29 |
|  |  | **% within diagnosis** | 51.2% | 9.5% | 5.0% | 20.0% |
| ***Pearson Chi-Square*** | | | *37.417* | | | |
| ***Likelihood Ratio*** | | | *35.290* | | | |
| ***Linear-by-Linear Association*** | | | *30.833* | | | |
| **OBS-SV88** | **Negative response** | **Count** | 34_a_ | 38_a, b_ | 57_b_ | 129 |
|  |  | **% within diagnosis** | 79.1% | 90.5% | 95.0% | 89.0% |
|  | **Positive**  **response** | **Count** | 9_a_ | 4_a, b_ | 3_b_ | 16 |
|  |  | **% within diagnosis** | 20.9% | 9.5% | 5.0% | 11.0% |
| ***Pearson Chi-Square*** | | | *6.613* | | | |
| ***Likelihood Ratio*** | | | *6.339* | | | |
| ***Linear-by-Linear Association*** | | | *6.212* | | | |
| **OBS-SV89** | **Negative response** | **Count** | 31_a_ | 38_a, b_ | 59_b_ | 128 |
|  |  | **% within diagnosis** | 72.1% | 90.5% | 98.3% | 88.3% |
|  | **Positive**  **response** | **Count** | 12_a_ | 4_a, b_ | 1_b_ | 17 |
|  |  | **% within diagnosis** | 27.9% | 9.5% | 1.7% | 11.7% |
| ***Pearson Chi-Square*** | | | *16.941* | | | |
| ***Likelihood Ratio*** | | | *17.296* | | | |
| ***Linear-by-Linear Association*** | | | *16.038* | | | |
| **OBS-SV90** | **Negative response** | **Count** | 33_a_ | 39_a, b_ | 56_b_ | 128 |
|  |  | **% within diagnosis** | 76.7% | 92.9% | 93.3% | 88.3% |
|  | **Positive**  **response** | **Count** | 10_a_ | 3_a, b_ | 4_b_ | 17 |
|  |  | **% within diagnosis** | 23.3% | 7.1% | 6.7% | 11.7% |
| ***Pearson Chi-Square*** | | | *7.860* | | | |
| ***Likelihood Ratio*** | | | *7.155* | | | |
| ***Linear-by-Linear Association*** | | | *6.069* | | | |
|  |  |  |  |  |  |  |
| **OBS-SV91** | **Negative response** | **Count** | 35_a_ | 38_a, b_ | 58_b_ | 131 |
|  |  | **% within diagnosis** | 81.4% | 90.5% | 96.7% | 90.3% |
|  | **Positive**  **response** | **Count** | 8_a_ | 4_a, b_ | 2_b_ | 14 |
|  |  | **% within diagnosis** | 18.6% | 9.5% | 3.3% | 9.7% |
| ***Pearson Chi-Square*** | | | *6.698* | | | |
| ***Likelihood Ratio*** | | | *6.785* | | | |
| ***Linear-by-Linear Association*** | | | *6.581* | | | |
| **OBS-SV92** | **Negative response** | **Count** | 30_a_ | 34_a_ | 60_b_ | 124 |
|  |  | **% within diagnosis** | 69.8% | 81.0% | 100.0% | 85.5% |
|  | **Positive**  **response** | **Count** | 13_a_ | 8_a_ | 0_b_ | 21 |
|  |  | **% within diagnosis** | 30.2% | 19.0% | 0.0% | 14.5% |
| ***Pearson Chi-Square*** | | | *19.480* | | | |
| ***Likelihood Ratio*** | | | *26.350* | | | |
| ***Linear-by-Linear Association*** | | | *18.979* | | | |
| **OBS-SV93** | **Negative response** | **Count** | 26_a_ | 36_b_ | 58_b_ | 120 |
|  |  | **% within diagnosis** | 60.5% | 85.7% | 96.7% | 82.8% |
|  | **Positive**  **response** | **Count** | 17_a_ | 6_b_ | 2_b_ | 25 |
|  |  | **% within diagnosis** | 39.5% | 14.3% | 3.3% | 17.2% |
| ***Pearson Chi-Square*** | | | *23.369* | | | |
| ***Likelihood Ratio*** | | | *23.611* | | | |
| ***Linear-by-Linear Association*** | | | *22.155* | | | |
| **OBS-SV94** | **Negative response** | **Count** | 33_a_ | 41_b_ | 60_b_ | 134 |
|  |  | **% within diagnosis** | 76.7% | 97.6% | 100.0% | 92.4% |
|  | **Positive**  **response** | **Count** | 10_a_ | 1_b_ | 0_b_ | 11 |
|  |  | **% within diagnosis** | 23.3% | 2.4% | 0.0% | 7.6% |
| ***Pearson Chi-Square*** | | | *21.609* | | | |
| ***Likelihood Ratio*** | | | *21.785* | | | |
| ***Linear-by-Linear Association*** | | | *17.875* | | | |
| **OBS-SV95** | **Negative response** | **Count** | 20_a_ | 24_a_ | 58_b_ | 102 |
|  |  | **% within diagnosis** | 46.5% | 57.1% | 96.7% | 70.3% |
|  | **Positive**  **response** | **Count** | 23_a_ | 18_a_ | 2_b_ | 43 |
|  |  | **% within diagnosis** | 53.5% | 42.9% | 3.3% | 29.7% |
| ***Pearson Chi-Square*** | | | *35.145* | | | |
| ***Likelihood Ratio*** | | | *41.992* | | | |
| ***Linear-by-Linear Association*** | | | *31.962* | | | |
| **OBS-SV96** | **Negative response** | **Count** | 17_a_ | 32_b_ | 59_c_ | 108 |
|  |  | **% within diagnosis** | 39.5% | 76.2% | 98.3% | 74.5% |
|  | **Positive**  **response** | **Count** | 26_a_ | 10_b_ | 1_c_ | 37 |
|  |  | **% within diagnosis** | 60.5% | 23.8% | 1.7% | 25.5% |
| ***Pearson Chi-Square*** | | | *45.655* | | | |
| ***Likelihood Ratio*** | | | *50.714* | | | |
| ***Linear-by-Linear Association*** | | | *44.526* | | | |
| **OBS-SV97** | **Negative response** | **Count** | 21_a_ | 35_b_ | 56_b_ | 112 |
|  |  | **% within diagnosis** | 48.8% | 83.3% | 93.3% | 77.2% |
|  | **Positive**  **response** | **Count** | 22_a_ | 7_b_ | 4_b_ | 33 |
|  |  | **% within diagnosis** | 51.2% | 16.7% | 6.7% | 22.8% |
| ***Pearson Chi-Square*** | | | *29.460* | | | |
| ***Likelihood Ratio*** | | | *28.713* | | | |
| ***Linear-by-Linear Association*** | | | *26.749* | | | |
| **OBS-SV98** | **Negative response** | **Count** | 22_a_ | 36_b_ | 58_b_ | 116 |
|  |  | **% within diagnosis** | 51.2% | 85.7% | 96.7% | 80.0% |
|  | **Positive**  **response** | **Count** | 21_a_ | 6_b_ | 2_b_ | 29 |
|  |  | **% within diagnosis** | 48.8% | 14.3% | 3.3% | 20.0% |
| ***Pearson Chi-Square*** | | | *33.623* | | | |
| ***Likelihood Ratio*** | | | *33.542* | | | |
| ***Linear-by-Linear Association*** | | | *30.833* | | | |
| **OBS-SV99** | **Negative response** | **Count** | 28_a_ | 38_b_ | 58_b_ | 124 |
|  |  | **% within diagnosis** | 65.1% | 90.5% | 96.7% | 85.5% |
|  | **Positive**  **response** | **Count** | 15_a_ | 4_b_ | 2_b_ | 21 |
|  |  | **% within diagnosis** | 34.9% | 9.5% | 3.3% | 14.5% |
| ***Pearson Chi-Square*** | | | *21.306* | | | |
| ***Likelihood Ratio*** | | | *20.380* | | | |
| ***Linear-by-Linear Association*** | | | *18.979* | | | |
| **OBS-SV100** | **Negative response** | **Count** | 25_a_ | 38_b_ | 58_b_ | 121 |
|  |  | **% within diagnosis** | 58.1% | 90.5% | 96.7% | 83.4% |
|  | **Positive**  **response** | **Count** | 18_a_ | 4_b_ | 2_b_ | 24 |
|  |  | **% within diagnosis** | 41.9% | 9.5% | 3.3% | 16.6% |
| ***Pearson Chi-Square*** | | | *29.033* | | | |
| ***Likelihood Ratio*** | | | *27.704* | | | |
| ***Linear-by-Linear Association*** | | | *25.196* | | | |
| **OBS-SV101** | **Negative response** | **Count** | 22_a_ | 29_a_ | 58_b_ | 109 |
|  |  | **% within diagnosis** | 51.2% | 69.0% | 96.7% | 75.2% |
|  | **Positive**  **response** | **Count** | 21_a_ | 13_a_ | 2_b_ | 36 |
|  |  | **% within diagnosis** | 48.8% | 31.0% | 3.3% | 24.8% |
| ***Pearson Chi-Square*** | | | *28.978* | | | |
| ***Likelihood Ratio*** | | | *33.428* | | | |
| ***Linear-by-Linear Association*** | | | *28.405* | | | |
| **OBS-SV102** | **Negative response** | **Count** | 27_a_ | 41_b_ | 59_b_ | 127 |
|  |  | **% within diagnosis** | 62.8% | 97.6% | 98.3% | 87.6% |
|  | **Positive**  **response** | **Count** | 16_a_ | 1_b_ | 1_b_ | 18 |
|  |  | **% within diagnosis** | 37.2% | 2.4% | 1.7% | 12.4% |
| ***Pearson Chi-Square*** | | | *34.577* | | | |
| ***Likelihood Ratio*** | | | *32.387* | | | |
| ***Linear-by-Linear Association*** | | | *26.474* | | | |
| **OBS-SV103** | **Negative response** | **Count** | 25_a_ | 40_b_ | 57_b_ | 122 |
|  |  | **% within diagnosis** | 58.1% | 95.2% | 95.0% | 84.1% |
|  | **Positive**  **response** | **Count** | 18_a_ | 2_b_ | 3_b_ | 23 |
|  |  | **% within diagnosis** | 41.9% | 4.8% | 5.0% | 15.9% |
| ***Pearson Chi-Square*** | | | *30.959* | | | |
| ***Likelihood Ratio*** | | | *28.470* | | | |
| ***Linear-by-Linear Association*** | | | *23.071* | | | |
| **OBS-SV104** | **Negative response** | **Count** | 27_a_ | 40_b_ | 60_b_ | 127 |
|  |  | **% within diagnosis** | 62.8% | 95.2% | 100.0% | 87.6% |
|  | **Positive**  **response** | **Count** | 16_a_ | 2_b_ | 0_b_ | 18 |
|  |  | **% within diagnosis** | 37.2% | 4.8% | 0.0% | 12.4% |
| ***Pearson Chi-Square*** | | | *35.081* | | | |
| ***Likelihood Ratio*** | | | *35.929* | | | |
| ***Linear-by-Linear Association*** | | | *29.659* | | | |

*Each subscript letter denotes a subset of diagnosis categories whose column proportions do not differ significantly from each other at the ,05 level.
