# Supplementary material for: Validation of the short version of the obsessive compulsive spectrum questionnaire
Source: Front Psychol. 2023 Jun 27;14:1157636. doi: 10.3389/fpsyg.2023.1157636 (PMC10333544; doi:10.3389/fpsyg.2023.1157636)
Supplement: Supplementary file 2 [file Table_2.docx]

**Supplementary Table 2**: Age descriptives

|  | | **Statistic** | **Std. Error** |
| --- | --- | --- | --- |
| **Mean** | | 39.42 | 1,070 |
| **95% C.I. for Mean** | Lower Bound | 37.31 |  |
|  | Upper Bound | 41.54 |  |
| **5% Trimmed Mean** | | 39.18 |  |
| **Median** | | 38.00 |  |
| **Variance** | | 165.968 |  |
| **Std. Deviation** | | 12.883 |  |
| **Minimum** | | 19 |  |
| **Maximum** | | 67 |  |
| **Range** | | 48 |  |
| **Interquartile Range** | | 21 |  |
| **Skewness** | | 0.288 | 0.201 |
| **Kurtosis** | | -1.079 | 0.400 |

**Supplementary Table 3**: Age descriptives divided for diagnosis

| **Diagnosis** | | | **Statistic** | **Std. Error** |
| --- | --- | --- | --- | --- |
| **DOC** | **Mean** | | 40.95 | 1.750 |
|  | **95% C.I. for Mean** | Lower Bound | 37.42 |  |
|  |  | Upper Bound | 44.49 |  |
|  | **5% Trimmed Mean** | | 40.83 |  |
|  | **Median** | | 40.00 |  |
|  | **Variance** | | 131.760 |  |
|  | **Std. Deviation** | | 11.479 |  |
|  | **Minimum** | | 20 |  |
|  | **Maximum** | | 64 |  |
|  | **Range** | | 44 |  |
|  | **Interquartile Range** | | 16 |  |
|  | **Skewness** | | 0.366 | 0.361 |
|  | **Kurtosis** | | -0.593 | 0.709 |
| **SAD** | **Mean** | | 40.95 | 2.040 |
|  | **95% C.I. for Mean** | Lower Bound | 36.83 |  |
|  |  | Upper Bound | 45.07 |  |
|  | **5% Trimmed Mean** | | 40.84 |  |
|  | **Median** | | 41.50 |  |
|  | **Variance** | | 174.827 |  |
|  | **Std. Deviation** | | 13.222 |  |
|  | **Minimum** | | 19 |  |
|  | **Maximum** | | 67 |  |
|  | **Range** | | 48 |  |
|  | **Interquartile Range** | | 24 |  |
|  | **Skewness** | | 0.075 | 0.365 |
|  | **Kurtosis** | | -1.215 | 0.717 |
| **HC** | **Mean** | | 37.25 | 1.740 |
|  | **95% C.I. for Mean** | Lower Bound | 33.77 |  |
|  |  | Upper Bound | 40.73 |  |
|  | **5% Trimmed Mean** | | 36.83 |  |
|  | **Median** | | 32.50 |  |
|  | **Variance** | | 181.614 |  |
|  | **Std. Deviation** | | 13.476 |  |
|  | **Minimum** | | 19 |  |
|  | **Maximum** | | 67 |  |
|  | **Range** | | 48 |  |
|  | **Interquartile Range** | | 23 |  |
|  | **Skewness** | | 0.504 | 0.309 |
|  | **Kurtosis** | | -1.082 | 0.608 |
