# Supplementary material for: Validation of the short version of the obsessive compulsive spectrum questionnaire
Source: Front Psychol. 2023 Jun 27;14:1157636. doi: 10.3389/fpsyg.2023.1157636 (PMC10333544; doi:10.3389/fpsyg.2023.1157636)
Supplement: Supplementary file 3 [file Data_Sheet_1.docx]

**OBS-SHORT VERSION**

Subject ID: ______________ Date:_________________

**Instructions:** We ask you to answer all the questions, taking into account the fact that not all of them refer to symptoms of illness, by ticking «No» or «Yes». Answer «Yes» even when you think only part of the question is true. _______________________________________________________________________________

**DOUBT**

**Were you often/ did you often feel…**

| **1** | … disturbed by constant doubts about the correct way of doing of daily activities, such as brushing your teeth, shaving, combing your hair, choosing the right outfit? | **Yes** | **No** |
| --- | --- | --- | --- |
| **2** | … very frustrated every time you didn't receive precise and immediate answers to your questions? | **Yes** | **No** |
| **3** | … impatient to know, as soon as a task or job is completed, if it was done correctly? | **Yes** | **No** |
| **4** | … disturbed by the lack of precise rules for doing everything? | **Yes** | **No** |
| **5** | … unable to stand your own opinions steadfastly because you were never absolutely sure that you were right? | **Yes** | **No** |
| **6** | … unprepared, or feared memory lapses, before an exam or any test, even if you knew the subject thoroughly? | **Yes** | **No** |
| **7** | … judged by all a touchy person? | **Yes** | **No** |
| **8** | … having difficulty choosing something on your own, without getting advice (for example which dress to wear, what to order at a restaurant, what to choose when making a purchase or whether to accept an invitation)? | **Yes** | **No** |
| **9** | … struggling to dispose of items, because they might be useful in the future? (e.g. used pens, previously read newspapers or magazines) | **Yes** | **No** |
| **10** | … unable to make a decision, because he tends to see the advantages and disadvantages of all alternatives at the same time and/or for fear of unpredictable consequences? | **Yes** | **No** |

**HYPERCONTROL**

**A. Caution**

**He has often been unwilling to…**

| **11** | … make changes in your daily habits and/or do something unexpected? | **Yes** | **No** |
| --- | --- | --- | --- |
| **12** | … doing something because he thought there was the possibility of an unfavorable outcome or out of fear that irrepressible worries or behaviors would be triggered (for example, touching a doorknob or refusing to shake someone's hand because then you would have to wash, or eat sweets because you would had to brush your teeth immediately afterwards)? | **Yes** | **No** |

**B. Control:**

**Have you ever felt compelled to…**

| **13** | … make memory efforts even when it would not have been necessary (for example remembering telephone numbers, car plates, dates and anniversaries)? | | **Yes** | **No** |
| --- | --- | --- | --- | --- |
| **14** | … check repeatedly … | |  |  |
|  | a | … to have closed the car doors, the gas, the doors or the lights of the house? | **Yes** | **No** |
|  | b | … shelves and drawers to make sure everything was in order? | **Yes** | **No** |
|  | c | … that you have not lost important or valuable objects (e.g. money, keys, documents, rings, bracelets)? | **Yes** | **No** |
|  | d | … that you have not made errors or omissions in those activities that require you to write (for example, rereading documents or assignments before submitting them, rereading or recopying letters, reopening and resealing envelopes before sending them)? | **Yes** | **No** |
|  | e | … the presence of dust or dirt? | **Yes** | **No** |
| **15** | … repeatedly check that you have not harmed yourself or others because of a sudden suspicion that you might have done so? | | **Yes** | **No** |
| **16** | … ask someone to check for you for fear of not being able to do it properly (e.g. asking neighbors or a passer-by to check if you have closed the door)? | | **Yes** | **No** |

**C. Emotional control**

**Were you ever, or has anyone ever told you that …**

| **17** | … you were unspontaneous, cold or detached towards others? | **Yes** | **No** |
| --- | --- | --- | --- |
| **18** | … you were hypercritical of himself and of others? | **Yes** | **No** |
| **19** | … you spoke and behaved ceremoniously and formally? | **Yes** | **No** |
| **20** | … you were humorless or unimaginative? | **Yes** | **No** |
| **21** | … you were very stubborn and/or had difficulty changing his point of view? | **Yes** | **No** |
| **22** | … you were incapable of getting involved in romantic relationships? | **Yes** | **No** |
| **23** | … you had difficulty letting go during sexual intercourse? | **Yes** | **No** |
| **24** | … you preferred to have sex according to established rituals or practices (for example, only at certain times, or in certain places) | **Yes** | **No** |

**Have you often encountered difficulties in…**

| **25** | … change the way you act and/or your methods of working, even when there could be a better system? | **Yes** | **No** |
| --- | --- | --- | --- |
| **26** | … adapt to the different character of people and/or work in team? | **Yes** | **No** |
| **27** | … grasp the nuances of things, tending to catalog according to "all-nothing", "black-white", "good-bad" schemes? | **Yes** | **No** |
| **28** | … lend and/or borrow clothes, books, records, or other personal items? | **Yes** | **No** |
| **29** | … entrust your children to a babysitter? | **Yes** | **No** |
| **30** | … spend money, so as to be judged stingy? | **Yes** | **No** |

**D. Other-directed control**

**Have you often felt the need to impose…**

| **31** | … your patterns and habits to family and friends (e.g., your sense of cleanliness, schedules)? | **Yes** | **No** |
| --- | --- | --- | --- |
| **32** | … your patterns and habits to employees and co-workers (e.g. office management, work planning, deadlines)? | **Yes** | **No** |
| **33** | … your political ideals, career choices, favorite sports to your family members? | **Yes** | **No** |
| **34** | Have you ever felt like you have to plan the lives of your family and friends and/or deal with their problems? | **Yes** | **No** |
| **35** | Have you ever felt challenged by your children and/or friends for your intrusive, overprotective or excessively controlling attitude? | **Yes** | **No** |

**E. Conformism and traditional values**

**Were you ever, or were you ever told that you…**

| **36** | … were too attached to tradition and/or were a firm believer in law and order? | **Yes** | **No** |
| --- | --- | --- | --- |
| **37** | … were unwilling to compromise on moral issues? | **Yes** | **No** |
| **38** | … were very involved in charitable or voluntary associations? | **Yes** | **No** |
| **39** | Do you have very classic tastes in the way you dress and in your hairstyle? | **Yes** | **No** |
| **40** | … were very attentive to the rules of etiquette? | **Yes** | **No** |
| **41** | Do you feel much fascination with military uniforms and/or do you tend to obey authority without objection? | **Yes** | **No** |
| **42** | Do you always keep your word? | **Yes** | **No** |

**F. Magical Thinking**

**Have you ever believed …**

| **43** | … in the possibility of changing the course of events through certain practices or rituals (for example by touching or arranging objects according to a certain procedure, pronouncing words, reciting magical formulas)? | **Yes** | **No** |
| --- | --- | --- | --- |
| **44** | … that the world was governed by fate or supernatural forces? | **Yes** | **No** |
| **45** | … to possess a certain 'sixth sense'? | **Yes** | **No** |
| **46** | … in horoscope, palm reading, evil eye, lucky or lucky numbers and/or colors? | **Yes** | **No** |
| **47** | … that something had happened, just because you have thought it or vice versa? | **Yes** | **No** |

**TEMPORAL DIMENSION**

1. **Time management**

**Have you often…**

| **48** | … used even the slightest scrap of time to devote yourself to study or work (for example during holidays, at the hairdresser's, in waiting rooms, waiting to start a meal, during commercial breaks)? | **Yes** | **No** |
| --- | --- | --- | --- |
| **49** | … preferred not linger at the table after eating but immediately start a new activity, even if you would have had time to relax? | **Yes** | **No** |
| **50** | … felt the need to fill your free time with productive activities (e.g. sport, study, work)? | **Yes** | **No** |

**Were you ever someone that, or were you ever told that you …**

| **51** | … take longer than his colleagues to complete his work, never being satisfied with the result? | **Yes** | **No** |
| --- | --- | --- | --- |
| **52** | … is often late for appointments because you lose track of time or can't finish previous commitments or don't want to risk waiting? | **Yes** | **No** |
| **53** | … is often early for appointments? | **Yes** | **No** |

**B. Slowness**

**Has anyone ever told you that you are …**

| **54** | … very slow in carrying out your usual daily activities (e.g. eating, talking, reading, writing, dressing, washing)? | **Yes** | **No** |
| --- | --- | --- | --- |

**PERFECTIONISM**

**Have you ever considered yourself …**

| **55** | … particularly tidy or precise? | **Yes** | **No** |
| --- | --- | --- | --- |
| **56** | … with poor synthetic ability in work or study, because you tend to get lost in the details? | **Yes** | **No** |
| **57** | … dissatisfied with your choices and results, despite your great precision and meticulousness (for example, you do not feel happy with the dress you are wearing, even after spending hours choosing that garment, or you are convinced that your house is dirty even after thorough cleaning )? | **Yes** | **No** |
| **58** | Do you have to copy the notes taken in lessons many times before starting to study? | **Yes** | **No** |
| **58** | Do you have to write a detailed list of all the things to do? | **Yes** | **No** |
| **60** | Must you have every object in the house, or in the office, always in the same exact position? | **Yes** | **No** |
| **61** | Do you still have to complete something, even if it is not necessary, or even when you are no longer interested in it (for example, finish reading a boring book before starting another)? | **Yes** | **No** |
| **62** | Do you have to delve into each topic in detail and consult encyclopedias, even for subjects of little importance? | **Yes** | **No** |
| **63** | Are you able to captures every detail of a house, a shop, a reception, an artistic event, a book or a document? | **Yes** | **No** |
| **64** | Do you collect valuables and non-valuables (e.g. stamps, postcards, coins, photographs, books, cooking recipes)? | **Yes** | **No** |
| **65** | Must you complete a collection, once the first copy has been purchased (for example series of books, magazines, dolls, comics, figurines)? | **Yes** | **No** |
| **66** | Do you keep even old clothes that he will almost certainly never wear again? | **Yes** | **No** |
| **67** | Do you keep addresses and telephone numbers of anyone you have ever known, due to the need to always have them on hand? | **Yes** | **No** |
| **68** | Do you systematically and regularly read newspapers or magazines? | **Yes** | **No** |
| **69** | Do you buy a book out of the need to have it, even if you have already read it? | **Yes** | **No** |
| **70** | Are you excessively attentive to the aesthetic form (symmetries, combination of colors, taste in dressing or furnishing)? | **Yes** | **No** |

**REPETITION AND AUTOMATION**

**Have you ever felt compelled to …**

| **71** | … repeat something until you are convinced that it is done the 'right' way (e.g. opening and closing a door, turning the light on and off, driving your car in and out of a parking space)? | **Yes** | **No** |
| --- | --- | --- | --- |
| **72** | … repeating a word several times, as if to improve its pronunciation or to give more emphasis to your speech and/or repeating a certain movement for no apparent reason and/or repeating a slogan or a musical tune, as if it doesn’t leave your mind? | **Yes** | **No** |
| **73** | … walk in a particular way? | **Yes** | **No** |
| **74** | … imitate the accent or the regional dialect of your interlocutor? | **Yes** | **No** |
| **75** | … touch an object or part of your body several times for no apparent reason? | **Yes** | **No** |
| **76** | … count repeatedly (e.g. add up number plates, pages in a book, windows or floors in a building)? | **Yes** | **No** |
| **77** | … make gestures, even if they are inconvenient or inappropriate, such as shouting, spitting, sniffing? | **Yes** | **No** |
| **78** | … clear your nose or clear your throat before starting to speak, make particular movements with your hand before starting to write or knock on the door? | **Yes** | **No** |

**OBSESSIVE THEMES**

**Have you ever felt haunted by thoughts, which you could not get rid of, concerning ...**

| **79** | … dirt, germs or contaminants and/or environmental pollutants? | **Yes** | **No** |
| --- | --- | --- | --- |
| **80** | … the fear of having eaten spoiled food or taken expired medicines? | **Yes** | **No** |
| **81** | … the presence in your body of toxic substances? | **Yes** | **No** |
| **82** | … the possibility of contracting infections using public toilets? | **Yes** | **No** |
| **83** | … the fear of contracting a disease, not necessarily contagious, for the mere fact of being near a sick person? | **Yes** | **No** |

**Have you ever felt compelled to ...**

| **84** | … have a particularly meticulous personal cleanliness (e.g. frequently washing hands, hair, teeth, carefully cleaning nails or taking a shower more than once a day)? | **Yes** | **No** |
| --- | --- | --- | --- |
| **85** | … demand from others a particularly meticulous personal cleanliness (for example from children or spouse)? | **Yes** | **No** |
| **86** | … require a meticulous cleaning of the house or car? | **Yes** | **No** |
| **87** | … repeatedly clean some parts of the house, but skipping others (e.g. cleaning the sink meticulously, but skipping the rest of the bathroom completely)? | **Yes** | **No** |

**Were you ever haunted by thoughts that you couldn't get rid of about ...**

| **88** | … your sexual identity? | **Yes** | **No** |
| --- | --- | --- | --- |
| **89** | … your sexual performance? | **Yes** | **No** |
| **90** | … scenes of intercourse or unusual or perverted sexual activity? | **Yes** | **No** |
| **91** | … the urge to look at your interlocutor's groin or to touch his genitals? | **Yes** | **No** |
| **92** | … the idea of sin? | **Yes** | **No** |
| **93** | … being able to think, say or perform inappropriate or even obscene acts in public? | **Yes** | **No** |
| **94** | … the need to atone for your alleged mistake or sin (for example by denying himself food, a moment of relaxation, a desired thing)? | **Yes** | **No** |
| **95** | … the future? | **Yes** | **No** |
| **96** | … the worry of not knowing how to start or maintain an important sentimental or friendship relationship? | **Yes** | **No** |
| **97** | … philosophical and existential themes (e.g. the meaning of existence, of the world, of God)? | **Yes** | **No** |
| **98** | … harming yourself (e.g. cutting yourself, jumping out of a window, balcony, under train or bus) and/or having the urge to do these things to someone else? | **Yes** | **No** |
| **99** | … hurting or killing someone (for example by handling knives, scissors or causing a fire or tampering with a safety device)? | **Yes** | **No** |
| **100** | … frightening or terrifying images or scenes relating to a traumatic event (e.g. accidents, disasters, deaths)? | **Yes** | **No** |
| **101** | … the fear of insulting other people? | **Yes** | **No** |
| **102** | … the fear of stealing or lying? | **Yes** | **No** |
| **103** | Have you ever become violent, aggressive or lost control because of your obsessions? | **Yes** | **No** |
| **104** | Has it ever happened to you to become violent, aggressive or lose control because others wanted to prevent you from carrying out your rituals, or because they did not comply with your requests? | **Yes** | **No** |

**Appendix I:**

**AGE RELATED MANIFESTATION**

**CHILDHOOD AND ADOLESCENCE**

**As a child or teenager do you remember, or were you ever told, that...**

| **1** | … you felt uncomfortable when friends or even relatives showed you their affection (for example, you refused kisses on the cheek from an early age)? | **Yes** | **No** |
| --- | --- | --- | --- |
| **2** | … you couldn’t enjoy yourself when you played, or did you not enjoy doing other things, because you were tormented by the idea of getting dirty or injured (for example, you did not enjoy playing sports, eating an ice cream cone, playing with sand)? | **Yes** | **No** |
| **3** | … you performed a series of fixed and pre-established actions at bedtime, before falling asleep (e.g. placed shoes, slippers, pillows, clothes, toys and books in a particular position, said good night sentences or prayers in a particular order and sequence; did you need to hear certain stories)? | **Yes** | **No** |
| **4** | … you were overly attentive to form and handwriting, or else you wrote in a particular and elaborate way? | **Yes** | **No** |
| **5** | … you were excessively attentive to the care of your notebooks, books and notes (for example you did not like that others touched them or asked to borrow them)? | **Yes** | **No** |
| **6** | … you memorized names, multiplication tables, historical dates, geographical notions (such as heights of mountains, lengths of rivers, number of inhabitants of cities and nations)? | **Yes** | **No** |
| **7** | … you pestered parents with a thousand questions about which animal was stronger, faster, or more aggressive and/or continually persisted with irresolvable questions about the 'why' and 'wherefore' of all things (e.g. the meaning or the origin of God and the world)? | **Yes** | **No** |
| **8** | … you thought you could never get things done in a way that would please your parents? | **Yes** | **No** |
| **9** | … you spent most of your free time in intellectual activities (for example reading, visits to museums or monuments, or topics concerning astronomy)? | **Yes** | **No** |
| **10** | … you were always looking for the perfect friend and were always dissatisfied with the ones you had? | **Yes** | **No** |
| **11** | … you were very jealous of your belongings and even of your friends? | **Yes** | **No** |
| **12** | … you were a particularly mature, responsible and disciplined child for your age? | **Yes** | **No** |
| **13** | … you felt very uncomfortable or guilty, when you did the smallest thing that didn't suit your family? | **Yes** | **No** |
| **14** | … you were very careful with clothing? | **Yes** | **No** |
| **15** | … you devoted a lot of time to your collections? | **Yes** | **No** |
| **16** | … you had a stutter and/or tics of some kind? | **Yes** | **No** |
| **17** | … you had the urge to kill and dissect animals to look at their internal organs? | **Yes** | **No** |
| **18** | … you did poorly in school because of these problems? | **Yes** | **No** |

**Appendix II:**

**ATYPICAL MANIFESTATIONS**

**IMPULSIVITY AND LOSS OF CONTROL**

**Have you ever felt compelled to …**

| **1** | … exercise excessively or follow a strict diet? | **Yes** | **No** |
| --- | --- | --- | --- |
| **2** | … lose control over drinking alcohol? | **Yes** | **No** |
| **3** | … lose control in eating up to binges? | **Yes** | **No** |
| **4** | … take all prescribed medications together? | **Yes** | **No** |
| **5** | … swear or say bad words, behaving in a completely unusual way for her? | **Yes** | **No** |
| **6** | … to gamble? | **Yes** | **No** |
| **7** | … make unnecessary and excessive purchases? | **Yes** | **No** |
| **8** | … pick and scratch to the point of skin lesions and hair pulling? | **Yes** | **No** |
| **9** | … masturbate repeatedly? | **Yes** | **No** |
| **10** | … commit petty theft? | **Yes** | **No** |
| **11** | … play with fire until you start fires? | **Yes** | **No** |
| **12** | … commit unlawful acts of a sexual nature? | **Yes** | **No** |

**Have you ever been haunted by thoughts about ...**

| **13** | … particular physical defects (e.g. the shape and/or size of the nose, genitals, legs, breasts, buttocks) so much that you repeatedly check yourself in the mirror and/or camouflage them (e.g. with your hands, with a particular hairstyle, with make-up, with clothing) and/or undergo cosmetic surgery? | **Yes** | **No** |
| --- | --- | --- | --- |
| **14** | … the worry of giving off bad smells (for example with sweat)? | **Yes** | **No** |
| **15** | … worry about having a serious physical illness (e.g. AIDS, cancer)? | **Yes** | **No** |
| **16** | … the idea that you could lose your hair and teeth? | **Yes** | **No** |
| **17** | … the idea that certain substances can permanently change your 'personality'? | **Yes** | **No** |
